# Evaluation of chimeric antigen receptor T cell therapy in non-human primates infected with SHIV or SIV

**DOI:** 10.1371/journal.pone.0248973

**Published:** 2021-03-22

**Authors:** Nami Iwamoto, Bhavik Patel, Kaimei Song, Rosemarie Mason, Sara Bolivar-Wagers, Cristina Bergamaschi, George N. Pavlakis, Edward Berger, Mario Roederer

**Affiliations:** 1 ImmunoTechnology Section, Vaccine Research Center, National Institutes of Allergy and Infectious Diseases, National Institutes of Health, Bethesda, Maryland, United States of America; 2 Human Retrovirus Section, Vaccine Branch, Center for Cancer Research, National Cancer Institute, National Institutes of Health, Frederick, Maryland, United States of America; 3 Laboratory of Viral Diseases, National Institutes of Allergy and Infectious Diseases, National Institutes of Health, Bethesda, Maryland, United States of America; Emory University School of Medicine, UNITED STATES

## Abstract

Achieving a functional cure is an important goal in the development of HIV therapy. Eliciting HIV-specific cellular immune responses has not been sufficient to achieve durable removal of HIV-infected cells due to the restriction on effective immune responses by mutation and establishment of latent reservoirs. Chimeric antigen receptor (CAR) T cells are an avenue to potentially develop more potent redirected cellular responses against infected T cells. We developed and tested a range of HIV- and SIV-specific chimeric antigen receptor (CAR) T cell reagents based on Env-binding proteins. In general, SHIV/SIV CAR T cells showed potent viral suppression in vitro, and adding additional CAR molecules in the same transduction resulted in more potent viral suppression than single CAR transduction. Importantly, the primary determinant of virus suppression potency by CAR was the accessibility to the Env epitope, and not the neutralization potency of the binding moiety. However, upon transduction of autologous T cells followed by infusion in vivo, none of these CAR T cells impacted either acquisition as a test of prevention, or viremia as a test of treatment. Our study illustrates limitations of the CAR T cells as possible antiviral therapeutics.

## Introduction

HIV infection is a chronic viral disease, generally treated with combination antiretroviral therapy to suppress HIV replication. However, virus infected cells can persist as a latent reservoir hidden from the immune system [1–3]. Latent reservoirs do not continuously produce virus but can do so upon cell activation [4]. To achieve a cure, eradication of the latent reservoir is necessary; this demands a high level of surveillance by self-renewing immune effector populations throughout the period of life-long latency.

Cytotoxic T lymphocytes (CTL) have the potential to kill and eradicate virus-infected cells [5]. However, CTL responses to HIV are diminished due to HLA/MHC-I down-regulation by the HIV Nef protein and the emergence of viral escape mutations [6, 7]. Targeting conserved regions on the viral protein can lower the possibility of selecting escape mutations which may present a fitness cost [8, 9]. CTL epitopes, however, are determined by the host HLA/MHC-I genotype, and is not easily translated to all susceptible indivivduals. An alternative to CTL is adoptive cell transfer (ACT) of expanded CTL. ACT has been developed for cancer therapy where it has achieved occasional regression of tumors. However, ACT has failed to show clinical benefits in HIV therapy [10].

In the rhesus macaque model for HIV, using SIV, adoptive transfer of SIV-specific CTL clones to chronically SIV-infected rhesus macaques failed to control viral replication, but gave an important insights for ACT [11]. In particular, the phenotype of *in vitro*-activated CTL clones skewed to terminal effector T cells. Those cells were trapped in the lung soon after infusion and did not traffic to lymph nodes where the virus was replicating. This study demonstrated the importance of T cell phenotype before infusion permitting the trafficking of transferred cells to intended sites [11].

An alternative to expanding autologous T cells ex vivo prior to re-infusion is to transfer T cells engineered to recognize targets through chimeric antigen receptors (CAR). Such cells, which overcome the requirement of MHC restriction, can be engineered with a variety of antigen-binding moieties including monoclonal antibodies (mAbs).

Here, we evaluated *in vitro* viral suppression activity by CAR T cells with different Env specificities, against either SHIV or SIV. Furthermore, we constructed dual-and triple-CAR transduced cells to increase the probability for detection of infected cells and to lower the possibility of escape mutations. In this NHP AIDS model, we compared persistence of CAR T cells expanded with different protocols to find an optimal condition to encourage transferred cells to traffic to sites of virus-replication. Finally, we assessed viral control after CAR T cell infusion into SHIV- or SIV-infected rhesus macaques to determine if CAR T cells can have a quantifiable impact on virus infected cells. Our study began with SHIV, using a potentially clinically-relevant technology; we then shifted to SIV to take advantage of a newly-available wide range of SIV-specific monoclonal antibodies [12]. Over all, we found no evidence for an impact on viral pathogenesis in these models.

## Results

### Evaluation of SHIV CAR-T cells in vitro

Previous studies used the HIV-Env binding domain of CD4 as a CAR targeting moiety, but no clinical benefits were observed upon adoptive transfer of CD4-CAR modified T cells [13]. However, the binding of this CD4-based CAR may not have had sufficient affinity. To improve the potency against HIV-Env, we created high-affinity bivalent CARs that consist of the gp120-binding domain of CD4 linked to a scFv of a CD4-induced epitope specific antibody, 17b. We compared the CD4 CAR to two CD4-17b CARs that varied in linker length between the CD4 and 17b domains; CD4-35-17b which contains a 35 AA linker long enough for simultaneous binding of both moieties to a single gp120 or CD4-10-17b which contains a 10 AA linker too short for simultaneous binding to the same gp120 subunit (S1 Fig).

**Fig 1:**
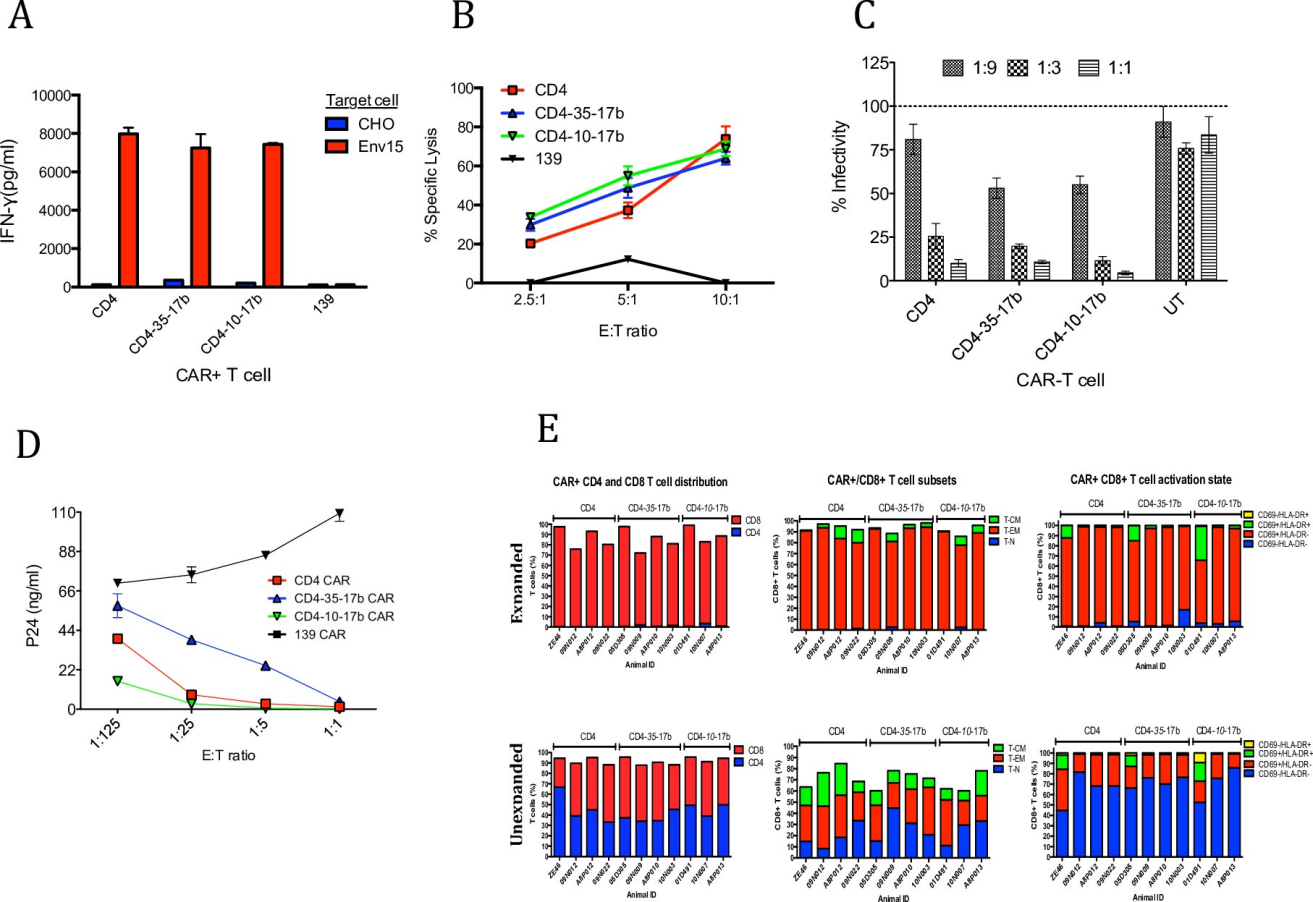
Viral suppression activity of anti-SHIV-CAR T cells in vitro and in vivo. (A) In vitro IFN-γ secretion assay evaluating immune activation of CAR-modified T cells against HIV-Env (IIIB) expressing cell line (Env15) and parental CHO control; all HIV-specific constructs display comparable IFN-γ secretion. (B) CD4-based CAR-modified T cells elicit specific, dose-dependent cytolytic activity against Env15 target cells. (C) CAR-mediated dose-dependent inhibition of HIV-1 (Ba-L) pseudotyped virus production by 293T cells transfected to produce HIV. (D) All CD4-based CAR+ T cells control spread of HIV (BX08) infection in a dose-dependent manner; CD4-10-17b CAR+ T cells display enhanced potency compared to the CD4 CAR in controlling the spread of infectious HIV within PBMCs whereas CD4-35-17b displayed reduced potency. (E) CAR-modified cells for adoptive transfer were either amplified ex vivo for 3 weeks (Expanded cells) or left unmodified (Unexpanded) prior to infusion. Expanded and unexpanded CAR-modified PBMC were mixed at a 2:1 ratio and ~2e8 total cells were adoptively transferred into SHIV-infected animals. Expanded CAR-modified PBMC (top) displayed a highly activated state and consisted primarily of CD8+ T-EM cells whereas unexpanded cells displayed modest activation and a more diverse distribution of T cell subsets. Error bars show 1SD of triplicate wells.

Initial in vitro experiments were performed to compare these constructs. In vitro activation assays using Env15 cells showed equivalent IFNγ secretion from CD4, CD4-35-17b and CD4-10-17b CAR T cells (Fig 1A). Non-specific activation was not observed on CHO cells. However, IFNγ secretion is not always concordant with killing activity; thus, we assessed direct killing activity using Env15 target cells. Here, we demonstrated a dose dependent increase in specific lysis of Env15 (Fig 1B). All anti-SHIV CAR T cells showed more than 50% specific lysis at E:T ratio of 10:1. We also tested viral suppression activity against HIV-1 (Ba-L) using pseudotype virus transfected in 293 T cells. In this assay, CD4-35-17b and CD4-10-17b demonstrated an increased viral suppression compared to CD4 CAR T cells (Fig 1C). Furthermore, we performed a viral suppression assay using replication competent HIV (BX08). In this assay, CD4-10-17b showed the most potent viral suppression and CD4-35-17b showed the least viral suppression (Fig 1D). Although all CD4-based CARs exhibited specific activity against HIV-1 gp120, CD4-10-17b displayed superior potency over CD4 and CD4-35-17b CARs in the context of inhibiting HIV-1 spread within human PBMC in vitro. Thus, we used this construct to test in vivo CAR transduction and activity against NHP infected with SHIV.

### Ex vivo SHIV CAR T cell preparation for in vivo study

We implemented SHIV CAR T cell experiments as shown in S2A Fig to evaluate CAR T cell efficacy. First, autologous CAR T cells were infused 8 weeks after SHIV-162P3 infection to test the impact on chronic infection, where virus was not suppressed by ART. In a second experiment, ART was initiated 2 weeks before CAR T cell infusion to assess if suppression of viremia might synergize to achieve viral control.

Based on earlier experiments demonstrating poor engraftment of highly expanded autologous CD8 T cells [11], we explored two different expansion techniques. We compared a 3 week in vitro expansion (“expanded”) for which a large number of highly differentiated cells could be obtained. This was compared to a no expansion after CAR transduction (“unexpanded”), resulting in a much smaller but relatively undifferentiated number of cells that should have better self-renewing capacity. The phenotype of CAR T cells prepared by each method were analyzed by flow cytometry (Fig 1E) and revealed that expanded cells were dominated by CD69^+^HLA-DR^−^effector memory (T_EM_) CD8 T cells (S3A Fig). In contrast, unexpanded CAR T cells maintained both CD4 and CD8 T cells and the proportion of T_EM_ was much lower (S3B Fig). Indeed, under the unexpanded protocol, 40% of these cells were resting memory (CD69^–^) cells.

### SHIV-CAR T cell transfer in NHP AIDS model

**Fig 2:**
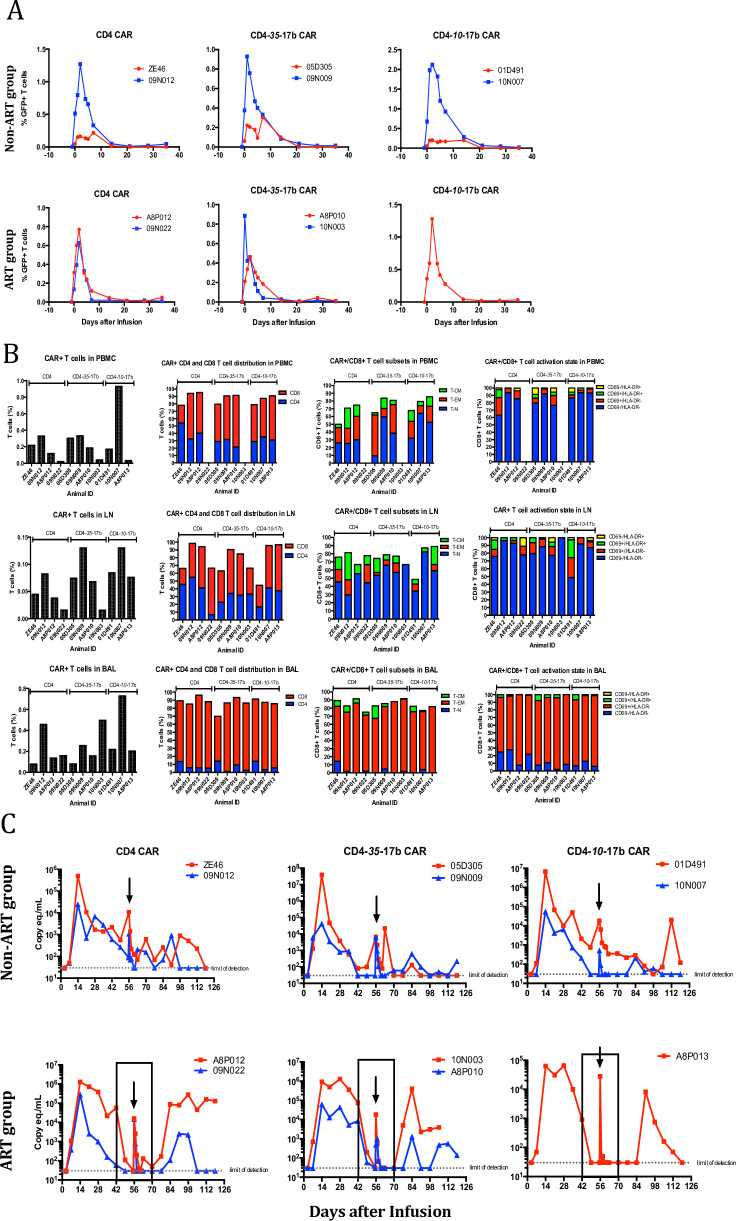
CAR T cell therapy on anti-SHIV-infected animals. (A) Adoptively transferred CAR-modified T cells were quantified within PBMC via flow-cytometry through the co-expressing fluorescent marker, eGFP, within non-ART treated animals (top row) and ART treated animals (bottom row). CAR+ T cells expand in PBMC within the first two days of adoptive transfer, but rapidly decline and become undetectable after 3 weeks. (B). CAR+ T cells were quantified and immunophenotyped within PBMC (top panel), inguinal lymph node (middle panel) and bronchoalveolar lavage (BAL, bottom panel) samples. Highly activated CD8+/CAR+ T cells traffic to the lung, likely deriving from the expanded cells from the infusion mixture whereas CAR+ cells within PBMC and LN tissue display the same phenotype as unexpanded cells from infusion mixture. *Insufficient CAR+ T cells were obtained for analysis. (C) Plasma viral load measurements indicate a high frequency of animals controlling SHIV infection. Measurements were taken on a weekly basis post-infection to monitor changes in viral loads and to determine if CAR+ T cell infusions exhibited antiviral activity. Arrows indicate time of CAR+ T cell adoptive transfer and the boxed areas in the lower panel designate the time points in which ART groups received therapy. The dramatic increase viral load at the time of infusion is from the transfer of autologous plasma containing SHIV RNA.

Expanded and unexpanded CAR T cells were mixed to determine which could persist better, and 2 x 10^8^ cells were transferred without further processing (or sorting) to SHIV-infected animals. CAR T cell frequencies in vivo were analyzed by flow cytometry based on eGFP expression after adoptive transfer. Although 2 x 10^8^ CAR T cells were infused, CAR T cells were detectable only for one week and none of CAR T cells were detected at 3 weeks post CAR T cell infusion (Fig 2A). The frequencies of CAR T cells in PBMC were less than 1% in T cells. CAR cells recovered from the LN were principally resting, whereas the BAL showed an accumulation of cells with an activated phenotype (Fig 2B). There was no obvious impact of CAR T cell transfer on viral loads (Fig 2C); the self-limiting nature of the viremia in SHIV models made discerning small effects impossible. The extremely transient viremia following infusion was due to the use of autologous plasma from an earlier, high-viral load time point used to resuspend the cells, and followed the kinetics expected of free virus persistence in plasma (6h half-life). The lack of a discernable impact on viral load may be due to the low, variable, and diminishing viremia common in SHIV-infected NHP, or the inability of the CD4-17b CAR T cells to persist or expand. Thus, we turned to the SIV model, for which more consistent pathogenesis is achievable, and for which NHP-native mAbs were available for construction of CARs.

### SIV neutralization by SIVmAbs

**Fig 3:**
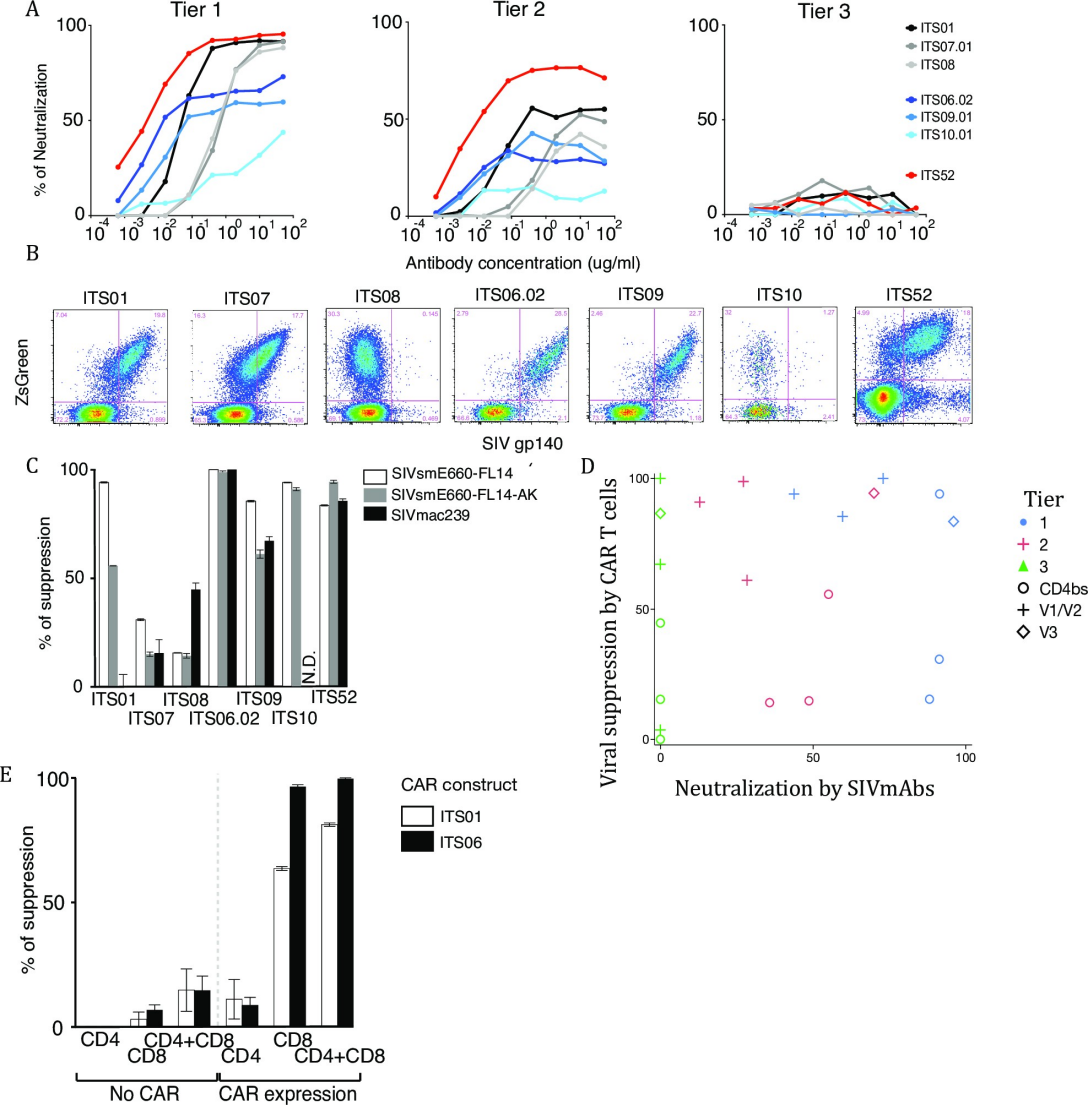
In vitro characterization of anti-SIV CAR. (A)Neutralization assay with original SIVmAb. Neutralization of Env-pseudotype virus by original SIVmAbs was measured on Tzm-bl cells. The graphs show neutralization of tier 1 (left), tier 2 (middle) and tier 3 SIV (right). The Y-axis shows percentage of neutralization and the X-axis shows antibody concentration. All assays were performed in duplicate. (B)Staining of anti-SIV-CAR T cells by SIVmac239 gp140 Env probe. SIV-CAR transduced rhesus PBMC were stained with SIVmac239 gp140 Env probe conjugated to PE. The Y-axis shows ZsGreen expression and the X-axis shows staining with the SIV gp140 probe. ZsGreen expression indicates CAR expression. (C)Viral suppression by sorted anti-SIV-CAR T cells. SIV-infected, activated rhesus CD8- cells were co-cultured with sorted anti-SIV-CAR T cells at 24 hrs after infection. CAR T cells were sorted based on ZsGreen expression and co-cultured with target cells at an E:T ratio of 1:1 in duplicate. Intracellular p27 expression was measured by flow cytometry. The percent of suppression was calculated based on a decrease in the percent of p27 positive cells compared with CAR T cell negative samples. Open bars show tier 1, grey bars show tier 2 and black bars show tier 3 virus suppression. ITS01, 07 and 08 are CD4bs, ITS06.02, 09 and 10 are V1/V2 and ITS52 is a V3-specific SIV-CAR. (D) Scatter plot between viral suppression by CAR T cells and virus neutralization by original SIVmAbs. No correlation between viral suppression by CAR T cells and virus neutralization by original SIVmAbs. (E) Viral suppression by CAR-negative and CAR T cells. ZsGreen negative and positive cells were sorted and used as effector cells in a viral suppression assay. ZsGreen negative cells were stained with CellTrace Violet to be distinguished from the target cells. All assays were performed in duplicate. The percent of suppression was calculated based on p27 expression in target cells. Open bars show suppression by ITS01 (CD4bs) and black bars show suppression by ITS06.02 (V1) CAR T cells.

We constructed seven anti-SIV CARs to test in the NHP AIDS model. We used three CD4 binding site (CD4bs)-targeting mAbs, three V1/V2-targeting mAbs, and one V3-targeting mAb. In terms of neutralization, the CD4bs and the V3-specific SIVmAbs completely neutralized tier 1 SIV; with tier 2 SIV, the V3-specific SIVmAb showed up to 80% neutralization, but the other SIVmAbs showed less than 60% neutralization. None of the SIVmAbs neutralized SIVmac239, which is tier 3 (Fig 3A).

### Anti-SIV CAR construction

We constructed single-chain Fv (ScFv) from these seven SIVmAbs and tested them as the antigen binding moieties of anti-SIV CARs. Anti-SIV CARs were constructed in a gamma retroviral vector (S1 Fig); reporter genes (ZsGreen and NGFR) were incorporated with an IRES, all under the regulation of a CD8α promoter. After transduction into rhesus T cells, anti-SIV CAR binding to SIV gp140 was confirmed by staining with PE-conjugated SIVmac239 gp140 Env trimer (Fig 3B). For the CD4bs anti-SIV CAR, ITS01 and ITS07 bound to the Env probe, but ITS08 did not show binding. For V1/V2 anti-SIV CARs, ITS06.02 and ITS09 showed strong binding to the Env probe. ITS10, a strain specific mAb, did not show binding to SVmac239 trimer but showed strong binding to SIVsmE660 gp120 monomers. ITS52 also strongly bound to SIV gp140. In general, the level of Env binding to transduced cells correlated with reporter gene expression.

### In vitro viral suppression activity by anti-SIV CAR T cells

We assessed viral suppression activity of anti-SIV CAR T cells with an *in vitro* viral suppression assay. SIV-infected rhesus CD8^–^ cells were co-cultured with sorted anti-SIV CAR T cells at an Effector to Target ratio at 1:1. Intracellular p27 in the target cells was stained on day 3 after co-culture. We tested viral suppression activity on SIV viruses (Fig 3C) that were categorized as tiers 1, 2 or 3 based on their neutralization susceptibility against multiple antibodies or polyclonal antisera. ITS01, V1/V2 and V3-specific anti-SIV CAR T cells showed more than 80% suppression on tier 1 SIV (SIVsmE660-FL14). However, viral suppression by ITS01 CAR T cells was less than 60% on tier 2 (SIVsmE660-FL14-AK), and 0% on tier 3 SIV (SIVmac239). ITS07 and ITS08 showed viral suppression under 50% on all SIV strains. On the other hand, ITS09, ITS10 and ITS52 showed equally potent viral suppression on tier 2 SIV as on tier 1 virus. Remarkably, ITS06.02 and ITS52 suppressed tier 2 and 3 SIV replication by more than 80%. Indeed, ITS06.02 completely suppressed all SIV strains. The viral suppression assay revealed that V1/V2 CAR T cells are the most potent, V3 CAR T cells are intermediate and CD4bs CAR T cells are the least potent in viral suppression. We conclude that the driving factor for CAR-mediated suppression was the targeted epitope, with no correlation between CAR-mediated suppression and neutralization (Fig 3D). Thus, epitope accessibility on the cell surface is key for construction of CAR T cells.

### Non-specific killing activity by CAR negative cells after transduction

Before attempting adoptive therapy in vivo, we assessed the need for purifying the CAR T cells from non-transduced (but stimulated) T cells. We sorted reporter-gene-expressing and non-expressing CD4 or CD8 T cells and co-cultured with SIV-infected CD8- cells. Non-specific killing was evaluated by the change in frequency of p27+ cells in the target cells (Fig 3E). Among the anti-SIV CAR T cell positive samples, CD8 T cells but not CD4 T cells showed viral suppression–however, the mixture of CD4 and CD8 was still fully suppressive. In CAR negative samples, viral suppression was not observed in any effectors. These results indicated that the main effector in viral suppression by anti-SIV CAR T cells were the CD8 T cells and we concluded that anti-SIV CAR T cell purification was not necessary before *in vivo* infusion, minimizing cell handling.

We evaluated the ability of SIV to infect CAR T cells in these cultures. Using flow cytometry to quantify p27+ cells as a measure of infection, we found that CD4+, but not CD8+ CAR T cells could be productively infected. In the co-cultures with both CD4 and CD8 CAR T cells, the frequency of infection of CD4 CAR T cells was reduced by 49% (N = 4) compared to cultures lacking CD8 CAR T cells.

### Multiple anti-SIV CAR transduction

**Fig 4:**
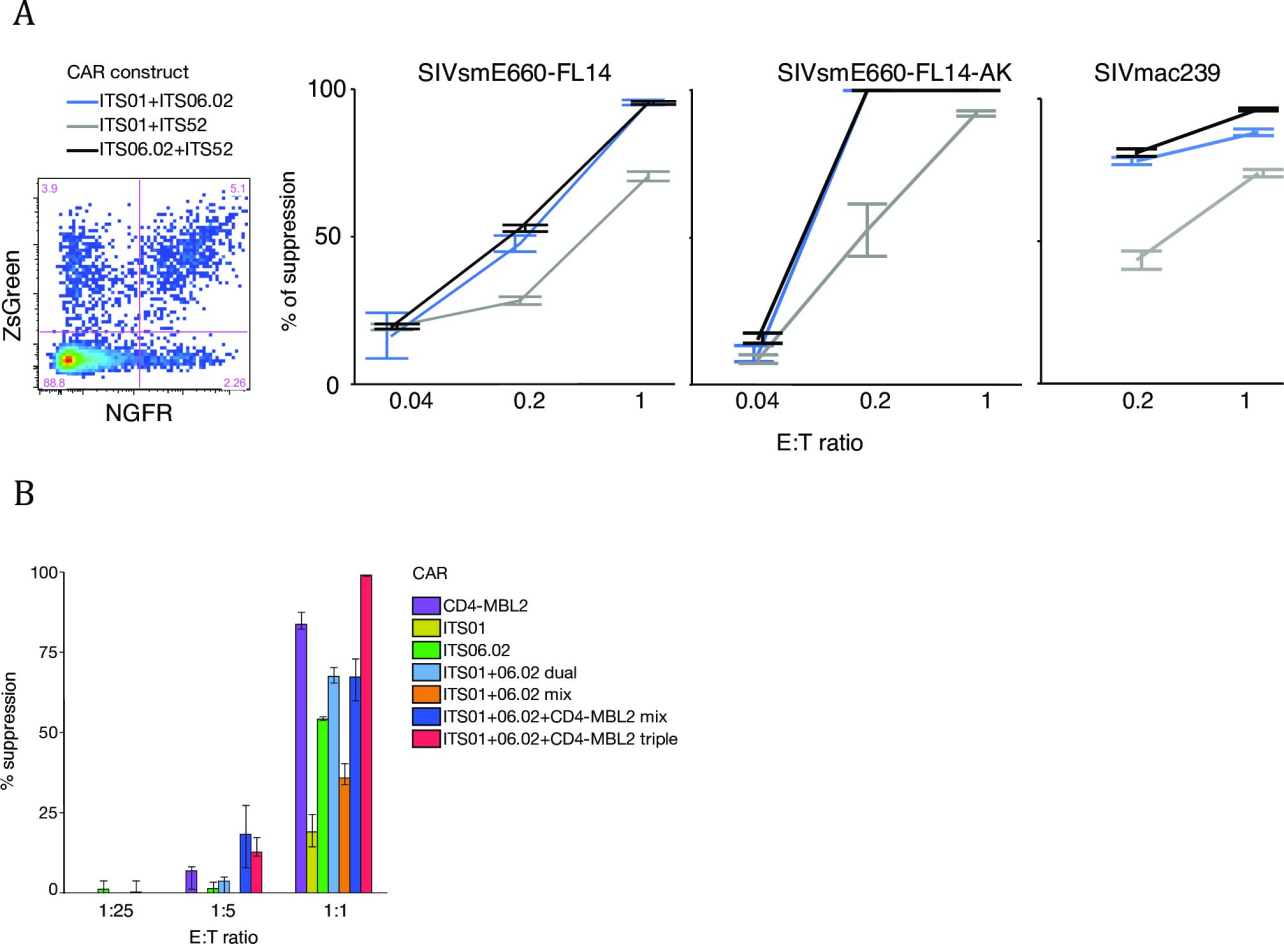
Viral suppression activity by dual and triple anti-SIV CAR T cells. (A) Dual anti-SIV-CAR transduction and viral suppression by dual anti-SIV-CAR T cells. Two different CAR vectors labeled with different reporter genes were mixed, coated on the same plates and transduced simultaneously. NGFR was stained with anti-NGFR-specific antibody and ZsGreen+NGFR+ cells were sorted for effector cells. Blue lines show suppression by ITS01 (CD4bs)+ITS06 (V1), grey lines show suppression by ITS01 (CD4bs)+ITS52 (V3) and black lines show suppression by ITS06.02 (V1)+ITS52 (V3) dual SIV-CAR T cells. Viral suppression on all tiers of SIV was assessed in duplicate. (B) Comparison of viral suppression efficacy based on valency. ITS01, ITS06.01 and/or CD4-MBL CAR were transduced to T cells separately or simultaneously. Single, dual or triple CAR T cells were sorted based on reporter gene or CAR expression. Sorted CAR T cells were co-cultured with SIVsmE660-FL14-infected cells in four replicates at E:T ratios of 1:1, 1:5 and 1:25. Percent reduction of p27-positive cells from CAR-negative samples were shown in the graph. E:T ratio at 0.04 was not performed for SIVmac239 due to shortage of CAR T cells for this assay.

Multiple antibody therapy and broad CTL responses are more effective for viral control compared to monotherapy. We compared dual anti-SIV CAR transduction with the expectation of a higher sensitivity for these cells to detect and kill infected cells, while simultaneously lowering the possibility of generating escape mutants. To make dual CAR T cells, two CAR constructs labeled with ZsGreen or NGFR were transduced into rhesus PBMC simultaneously and dual anti-SIV CAR T cells were identified by co-expression of the both reporter genes (Fig 4A). Sorted dual anti-SIV CAR T cells and target cells were co-cultured at E:T ratios of 1:1, 1:5 and 1:25. Dual anti-SIV CAR T cells expressing ITS06.02 showed similar potency regardless of the combination and viral suppression reached more than 50% on all tiers of SIV even at an E:T ratio of 1:5. The fraction of viral suppression was higher by dual anti-SIV CAR T cells than by single anti-SIV CAR T cells. However, the combination of ITS01 and ITS52 was not as potent as other combinations. Based on these in vitro data, we chose to combine ITS01 and IS06.02 for therapeutic studies in an NHP AIDS model.

In later experiments, we further attempted triple CAR T cell transduction by including a simianized CD4-MBL CAR that targets the CD4 binding site and glycan on SIV envelope (S4 Fig). We compared viral suppression efficacy between multiple anti-SIV CAR T cells and mixed single anti-SIV CAR T cells. Interestingly, multiple CAR-transduced T cells demonstrated better viral suppression activity than mixture of single CAR-transduced T cells. These data indicated that co-expression of distinct anti-SIV CAR molecules on the cell surface resulted in higher viral suppression efficacy.

### Comparison of in vitro CAR T cell expansion methods before in vivo infusion

In cancer immunotherapy, CAR T cells are expanded with CD3 and CD28 stimulation for a few weeks before *in vivo* transfer. However, it is known that strong stimulations and long-term *in vitro* culture skew CAR T cell phenotype to effector memory. Effector memory T cells circulate in the blood but may not traffic to lymph nodes from circulation due to lack expression of CD72L and CCR7. In HIV therapy, we hypothesize that CAR T cells need to traffic to lymph nodes, a major site of viral replication. To find an optimal *in vitro* culture condition that enhances CAR T cells trafficking to lymph nodes and persist longer, we prepared CAR T cells using two different protocols and compared tissue trafficking and *in vivo* persistence after infusion. First, based on clinical protocols, CAR T cells were expanded for one month in the presence of feeder cells. For our second protocol, after transduction, CAR T cells were cultured with IL-2 (200 IU/ml) for 4 days. By this method the CAR T cell phenotype remains relatively undifferentiated and similar before and after transduction.

**Fig 5:**
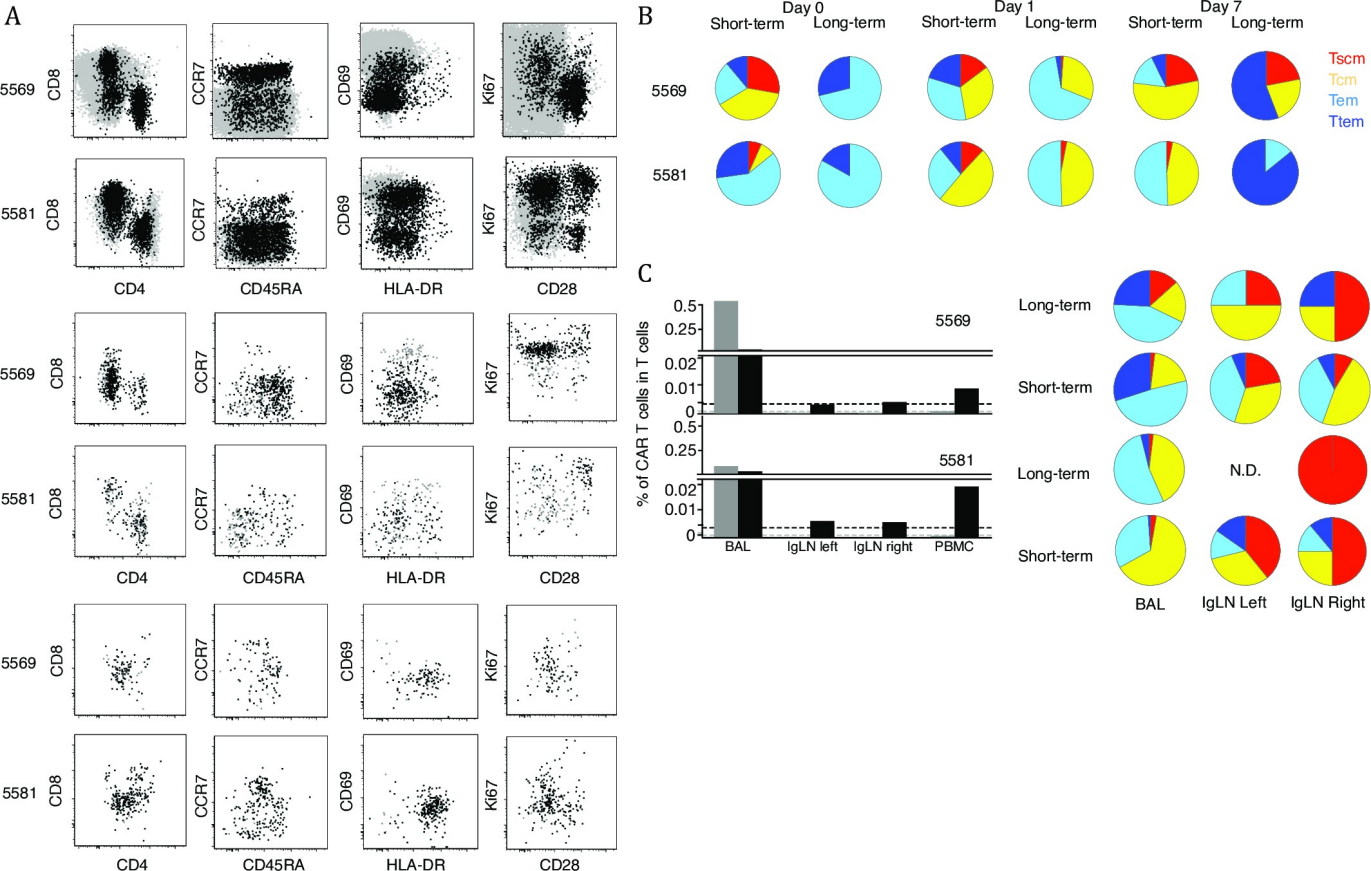
Comparison of CAR T cell expansion protocols in vivo. (A) Flow plot of anti-SIV-CAR T cells on the day of infusion and on 1 and 7 days after infusion. SIV-CAR T cells were gated based on reporter gene expression. CAR T cell phenotypes were analyzed by the expression of CD3, CD4, CD8, CD28, CD45RA, CD69, CD95 and CCR7. Ki67 was stained to check the in vivo proliferation of SIV-CAR T cells. (B) Anti-SIV-CAR T cell memory phenotypes SIV-CAR T cell memory phenotypes were classified based on CCR7 and CD45RA expression. (C)Tissue trafficking of anti-SIV-CAR T cells. Frequencies of anti-SIV-CAR T cells were measured on day 7 after infusion in BAL and inguinal lymph nodes. Grey bars show the percent of long-term expanded SIV-CAR T cells and black bars show the percent of short-term expanded anti-SIV-CAR T cells in CD3+ T cells. Pie charts show memory phenotypes of anti-SIV-CAR T cells in each tissue.

Autologous CAR T cells expanded by the two methods were mixed and transferred to the same rhesus macaques (S2B Fig). The long-term expansion favors CD8 T cells over CD4 T cells; similarly, the cells were uniformly CCR7^–^CD28^–^ and CD69^+^ T_EF_ cells (Fig 5A). In contrast, the short-term expanded CAR T cells consisted of both CD4 and CD8 T cells and CCR7 and CD28 expression were maintained. Not all cells were CD69^+^, suggesting the short-term culture could maintain a less activated phenotype than the long-term culture.

On the day of infusion, long-term expanded CAR T cells only had effector and terminal effector memory phenotypes (Fig 5B). Short-term expanded CAR T cells had stem cell memory and central memory phenotypes that can traffic to lymph nodes. On day 1 after infusion, the ratio of terminal effector memory T cells transiently decreased for long-term expanded CAR T cells, but the majority of CAR T cells on day 7 were terminal effectors while ratio of central memory T cells increased in the transfused short-term expanded CAR T cells. This result suggests that the long-term expanded CAR T cells became exhausted soon after infusion, but short-term expanded CAR T cells had more potential to proliferate in response to SIV-infected cells.

We examined tissue trafficking to Bronchoalveolar lavage (BAL) and inguinal lymph nodes on day 7 after infusion. When more than 70 million long-term expanded CAR T cells were infused, their frequency was below the background level in PBMC and inguinal lymph nodes; the BAL contained high frequency of long-term expanded CAR T cells. On the other hand, short-term expanded CAR T cells were found in the BAL, but also detected at inguinal lymph nodes and PBMC. Phenotypic analysis showed that the majority of CAR T cells found in the BAL had an effector memory phenotype.

These data are consistent with adoptively transferred effector T cells preferentially being trapped in the lung, as was previously seen with autologous expanded T cell clones [11] (Fig 5C). The major memory phenotypes detected in inguinal lymph nodes were stem cell memory and central memory. This suggests that keeping stem cell memory and central memory phenotypes is important for trafficking to the lymph nodes [14].

### Anti-SIV CAR T cell therapy in an NHP AIDS model

**Fig 6:**
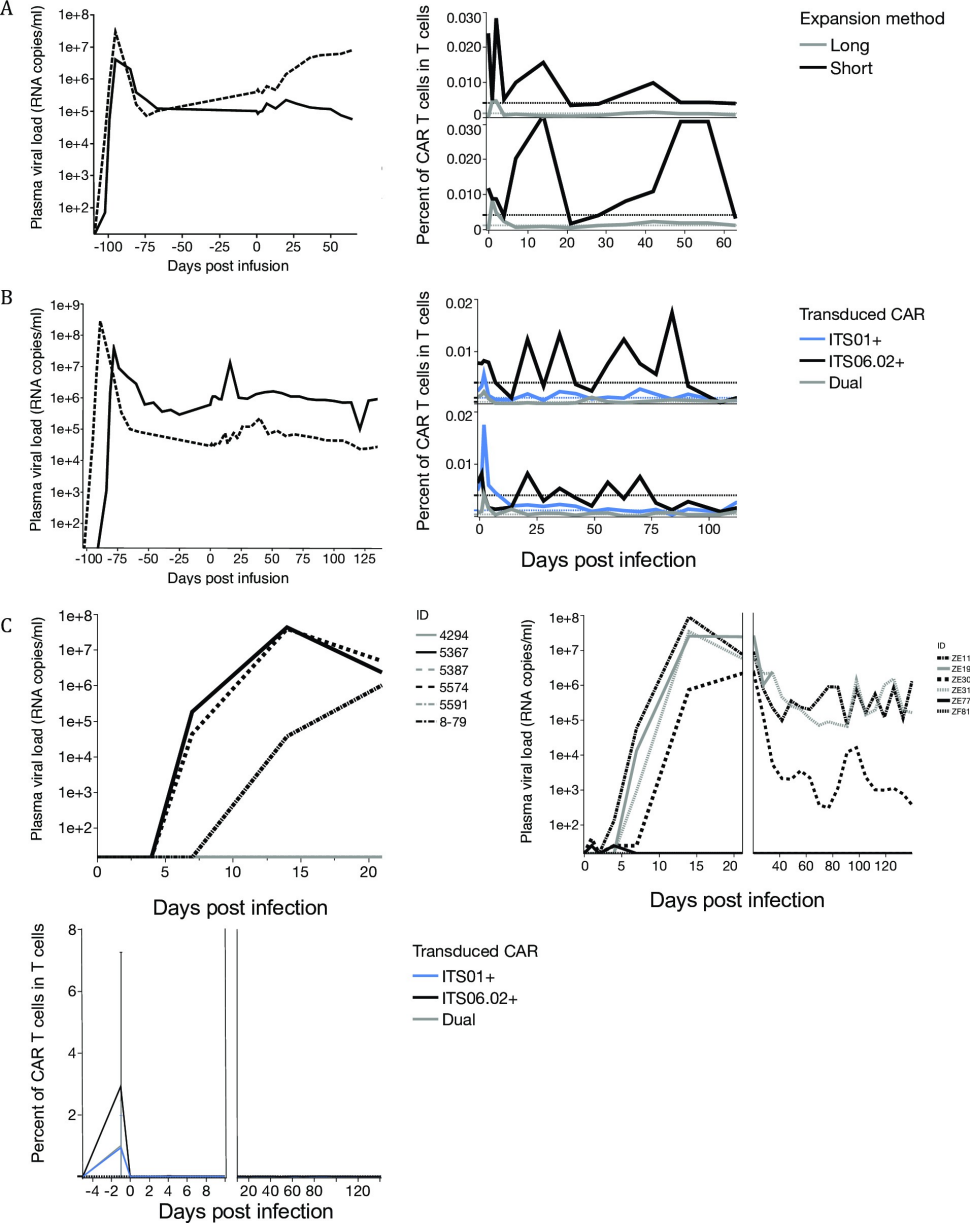
In vivo persistence of anti-SIV-CAR T cells and impact on plasma viral loads. (A)Plasma viral load and long- and short-term expanded SIV-CAR T cell persistence in chronically SIV-infected rhesus macaques. Left graph shows plasma viral loads before and after SIV-CAR T cell infusion. Right graph shows frequency of SIV-CAR T cells in T cells in PBMC. Black lines show frequencies of short-term expanded SIV-CAR T cells and grey lines show those of long-term expanded SIV-CAR T cells. Dashed lines show background level of reporter gene expression in SIV-CAR negative rhesus macaques. (B) Plasma viral loads and SIV-CAR T cell in chronically SIV-infected rhesus macaques. Left graph shows plasma viral loads before and after SIV-CAR T cell infusion. Right graph shows SIV-CAR T cell persistence in T cells in PBMC. Black lines show the frequency of ITS01, grey lines show ITS06 and blue lines show that of dual SIV-CAR T cells. (C) Plasma viral loads and persistence of SIV-CAR T cells in SIV-CAR prevention study. Left graphs show plasma viral loads of rhesus macaques in the control group and right graph is those of SIV-CAR–treated group. Bottom graph shows SIV-CAR T cell persistence in T cells in PBMC. Black lines show the frequency of ITS01, grey lines show ITS06 and blue lines show that of dual SIV-CAR T cells.

Plasma viral loads and anti-SIV-CAR T cell persistence were assessed in the cell expansion method comparison study, in which animals were infected with the tier 2 clone SIVsmE660-FL14-AK three months prior. Fig 6A shows plasma viral loads and the frequency of anti-SIV CAR T cells in peripheral T cells; there was no discernable impact on plasma viral loads. The frequency of long-term expanded anti-SIV CAR T cells in peripheral T cells was below the background level throughout the study, but the frequency of short-term expanded T cells fluctuated, being detectable on occasion for months. Because of the *in vivo* persistence and tissue trafficking, we decided to apply the short-term cell expansion protocol to future studies.

We then treated chronically SIVsmE660-FL14 infected rhesus macaques with ITS01 and ITS06.02 dual anti-SIV CAR transduced cells (Fig 6B, S2C Fig). Using the short-term culture method, the total numbers of cells infused were 700 thousand (animal 5572) and 44 million (animal 5573), respectively. Dual SIV-CAR T cells showed more potent viral suppression *in vitro*, but this did not translate to an impact on viral loads after CAR T cell infusion. In SIV-negative animals, ITS01-CAR T cells persisted less than 75 days and the frequencies of ITS06.02 and ITS52-CAR T cells went below the background level by 50 days post infusion. In SIV-infected animals, ITS01 and ITS06.02 persisted more than 50 days. This result shows antigen-specific expansion and persistence in SIV-infected animals, but not to levels that could impact viral replication.

### Evaluation of the impact of anti-SIV-CAR T cell infusion on acute SIV infection

We assessed the impact of CAR T cell transfer on acute SIV-infection to determine if the CAR T cells could impact pathogenesis when viremia was minimal. In this study, autologous ITS01 and ITS06.02 transduced anti-SIV-CAR T cells were transferred to six SIV negative animals and those animals were challenged with SIVsmE660 intrarectally at a limiting challenge dose of 10 TCID_50_ on the next day of CAR T cell transfer (S2D Fig). Six animals were challenged with SIVsmE660 at the same dose as a control group. In the control group, three of six animals became infected (Fig 6C). In the anti-SIV-CAR T cell treated group, four out of six animals became infected. One animal showed less than 10^4^ copies/ml of plasma viral load at the setpoint, but this animal had MamuA01 and likely its intrinsic CTL response contributed to the low viral load. Neither CAR T cell persistence nor expansion was observed in CAR-treated animals.

### Triple CAR T cell therapy

**Fig 7:**
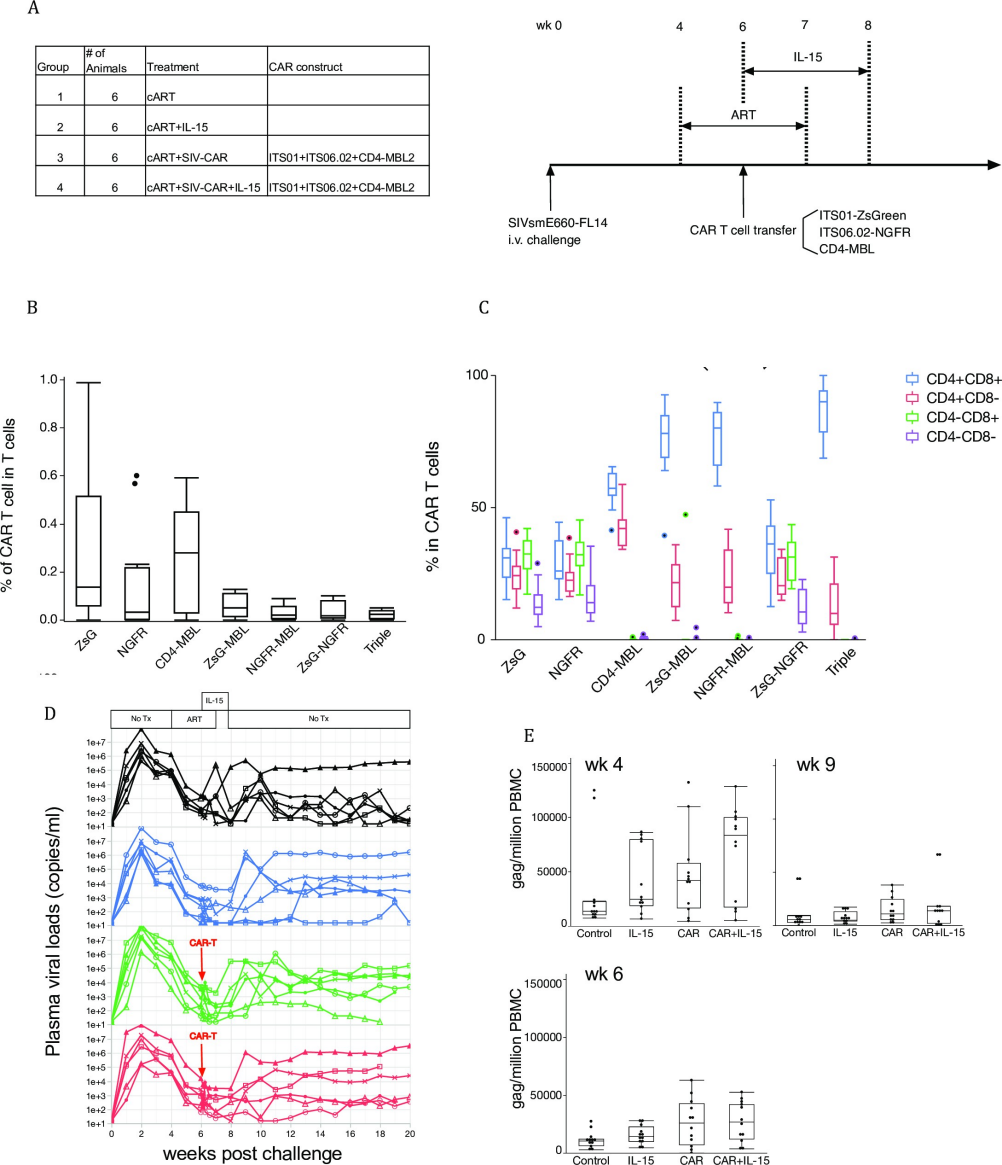
Combination therapy by CAR+IL-15. (A) Study groups of CRA+IL-15 study. Anti-SIV-CAR T cells were prepared from PBMC before infection and 1 week before CAR T cell transfer. ART started 2 weeks before CAR T cell transfer and continued for 3 weeks. IL-15 injection was initiated on the same day of CAR T cell transfer and continued for 2 weeks. (B) Percent of single, dual and triple CAR T cells before in vivo transfer. (C) CD4 and CD8 expression on CAR T cells. Bar and whisker plots are Tukey outlier plots show the median, IQR, and outliers, (D) Plasma viral loads in each group. Black line: Control, Blue line: IL-15, Green line: CAR and Red line: CAR+IL-15. ART period is shaded with grey and CAR transfer is indicated as a red dashed line. The period of IL-15 therapy is shown with an arrow. (E) Proviral load in PBMC. Gag copy number per million cells were calculated and shown in the graph.

We hypothesized that IL-15, a mitotic cytokine for memory CD8 T cells, might improve the expansion and durability of CAR T cells. We initiated this study using triple CAR T cells (Fig 4B) to generate the most sensitivity for virus-infected cells. We enrolled four groups in this study: control, IL-15 treatment alone, CAR therapy alone, and IL-15 and CAR therapy, as shown in Fig 7A. In addition, we treated all groups with ART from wk 4 to 8 post challenge. Anti-SIV CAR T cells were transferred at wk 6 and IL-15 treatment started simultaneously and continued until wk 8.

The proportion of single CAR T cells were higher than dual and triple CAR T cells before infusion (Fig 7B). Notably, the memory phenotypes that had the highest transduction efficiency were T_CM_ and T_EM_ (S5 Fig). We collected lymph node biopsies at wk 6, 7, 8 and 12, and CAR T cells were low but detectale in lymph nodes as well as in blood (Table 1). We expected CAR T cells would have expanded by IL-15 injection, but no impact was seen on the frequency of CAR T cells nor the memory phenotypes of CAR T cells (S6A Fig). We could not detect most CAR T cell combinations in LN or blood at later time points. On the other hand, CAR T cells were detected in BAL even at wk 12, when CAR T cells were not detected in PBMC (Table 2). These results suggest that IL-15 treatment improved persistence in BAL, but it did not help trafficking to virus replication sites nor with CAR T cell expansion. IL-15 stimulates CD8 T cells, so we determined the balance of CD4 and CD8 CAR T cells before transfer. Dual and triple CAR were mainly transduced in CD8 T cells (CD4^+^CD8^+^ and CD4^–^CD8^+^ in Fig 7C).

**Table 1:** CAR T cell frequency in PBMC.

Weeks post challenge	CAR T cell	CAR	CAR+IL-15
6	ZsG	0.0052	0.0133
6	ZsG-NGFR	0.0004	0.0013
6	NGFR	0.0131	0.0334
6	CD4-MBL	0.0707	0.0941
6.1	ZsG	0.0040	0.0114
6.1	ZsG-NGFR	0.0000	0.0000
6.1	NGFR	0.0292	0.0203
6.1	CD4-MBL	0.1285	0.1465
6.6	ZsG	0.0023	0.0053
6.6	ZsG-NGFR	0.0000	0.0000
6.6	NGFR	0.0211	0.0069
6.6	CD4-MBL	0.1035	0.1265
7	ZsG	0.0034	0.0024
7	ZsG-NGFR	0.0000	0.0000
7	NGFR	0.0183	0.0072
7	CD4-MBL	0.0903	0.1495
8	ZsG	0.0009	0.0019
8	ZsG-NGFR	0.0000	0.0000
8	NGFR	0.0175	0.0063
8	CD4-MBL	0.0848	0.0959
9	ZsG	0.0011	0.0016
9	ZsG-NGFR	0.0002	0.0001
9	NGFR	0.0091	0.0082
9	CD4-MBL	0.1007	0.0717

**Table 2:** CAR T cell frequency in BAL.

Weeks post challenge	CAR T cell	CAR	CAR+IL-15
7	ZsG	0.0048	0.0149
7	ZsG-NGFR	0.0011	0.0021
7	NGFR	0.0340	0.0613
7	CD4-MBL	0.3095	0.4705
8	ZsG	0.0047	0.0162
8	ZsG-NGFR	0.0000	0.0021
8	NGFR	0.0329	0.0494
8	CD4-MBL	0.2525	0.2840
12	ZsG	0.0080	0.0090
12	ZsG-NGFR	0.0017	0.0032
12	NGFR	0.0275	0.0475
12	CD4-MBL	0.3135	0.2900

Increased plasma IL-18 concentrations were previously identified as biomarker of IL-15 activity [15–18]. We determined the circulating IL-18 levels during hetIL-15 administration following step-dose regimen. Before treatment, the basal level of plasma IL-18 in rhesus macaque averaged 80 pg/ml (S6B Fig). A single injection of hetIL-15 was sufficient to elicit a 2-fold increase in the level of plasma IL-18, measured at day 4 (p<0.01, One-way Anova). In animals treated with the hetIL-15 step-dose regimen, we observed a progressive increase in plasma IL-18 levels throughout the treatment with peak levels at day 21 (3-fold increase, p = 0.02, One-way Anova). Based on these data, we confirmed that hetIL-15 demonstrated bioactivity on immune system.

We saw the expected rapid SIV control soon after ART, but viral load did not drop completely with 4-week ART. Some animals spontaneously control virus and we could not determine a significant effect of CAR T cell therapy on either plasma viral load or DNA viral load (Fig 7D and 7E).

## Discussion

To overcome HLA restriction in CTL responses, chimeric antigen receptors (CAR) have been developed for cancer therapy [19]. CARs consist of a target-binding moiety coupled to the intracellular domain of co-stimulatory molecules and the CD3 zeta chain. Gammaretrovirus and lentivirus vectors have been used for CAR transduction to T cells.

In clinical studies, CAR-transduced T cells (CAR T cells) are expanded by TCR stimulation then transferred to patients. CD19 CAR (B cells) has achieved remission of lymphoma [20, 21]. However, CAR T cell infusion can cause systemic inflammatory response resulting in organ damage or death [22]. Therefore, transferring billions of CAR T cells is not always favorable even though a higher number of effector cells are advantageous for killing target cells.

CAR also has been developed for HIV therapy. A main target of HIV-CARs is the CD4 binding site (CD4bs) of the envelope protein (Env) expressed on virus-producing cells. Although long-term survival of HIV-specific CAR T cells *in vivo* was reported, CAR T cell transfer did not result in viral control among HIV patients [23]. CAR T cell transfer needs to be improved to achieve a complete cure, which includes eradication of viral reservoirs.

In order to achieve viral control by CAR T cells, we focused on three points in this study: the targeted moiety, the ex vivo CAR T cell expansion method, and the in vivo trafficking of transferred CAR T cells.

As reported previously, CD4-35-17b demonstrated stronger viral suppression than CD4 or CD4-10-17b in vitro [24]. For preparation of SHIV-CAR T cells, we compared two methods; one including dramatic expansion (with concomitant differentiation) of cells, and one with minimal expansion. Expanded CAR T cells had effector memory phenotype and unexpanded CAR T cells maintained resting phenotype. Expanded and unexpanded CAR T cells were mixed at 2:1 ratio, but majority of CAR T cell phenotype detected in PBMC and lymph nodes were CD69^−^HLA-DR^−^. On the other hand, CD69^+^HLA-DR^+^ activated CAR T cells were detected mainly in BAL as shown in the previous study [11]. This result indicates that unexpanded CAR T cell phenotype are optimal for tissue circulation. However, CAR T cell efficacy was not detectable, likely due to the variable and spontaneous viral control in SHIV AIDS model.

We turned to an SIV AIDS model that has consistent pathogenicity. We constructed CAR using SIV-specific antibodies, as a CD4-based CAR was developed and tested in a clinical study, but did not show any impact on plasma viral load in spite of long-term persistence [13, 23]. We first tested anti-SIV-CAR with different specificities (S4 Fig). We compared viral suppression by CD4bs, V1/V2 and V3-specific CAR T cells and found that V1/V2-specific CAR had the most potent and consistent viral suppression. While HIV-specific CARs were constructed using broad bNAbs, and CD4bs-specific CAR were the most potent [25], we found that accessibility on the cell surface was paramount. Our in vitro data showed that V1/V2-specific CAR were superior to CARs targeting the CD4bs and V3 glycan on the SIV Env.

We compared the activity of dual- and triple-CAR transduction for SIV CAR for killing infected cells. We hypothesized that these multiply-transduced CAR T cells would have more sensitive detection of virally-infected cells even if escape mutations to one of the antibodies appeared. Indeed, we found that additional CAR targeting moieties on each cell improved viral suppression. We also tested a simianized CD4-MBL bi-specific CAR that targets glycan and CD4bs. CD4-MBL CAR exhibited the most potent viral suppression. Based on our data, targeting multiple site leads to potent viral suppression as reported in cancer therapy [26]. This concept has recently been proven by anti-HIV duoCAR-T cells [27]. In line with our study, neutralization resistance did not correlate with viral control.

In an in vivo study, we optimized CAR T cell expansion methods. Latently HIV-infected cells reside in lymph nodes [28]. Accessibility to virus-infected cells in lymph nodes would be a determinant for viral control. Our short-term expansion method successfully maintained CCR7 expression that is a trafficking marker to lymph nodes. Despite better targeting of lymph nodes with short-term expansion, CAR-T cells did not have any discernable impact on plasma viral load nor proviral load.

Since it is difficult to make large numbers of CAR T cells by short-term culture in vitro, we combined CAR therapy with cytokine therapy with the aim to induce expansion of CAR T cells in vivo. IL-15 is a cytokine that is required for homeostatic maintenance of long-lived CD8 memory T cells [29]. Furthermore, it is reported that tumor remission by anti-CD19 CAR associated high serum IL-15 levels in cancer therapy [30]. We hypothesized that a combination of IL-15 and CAR therapy might trigger expansion of T_SCM_ and T_CM_ and facilitate trafficking of CAR T cells to lymph nodes. However, CAR T cells were only detected for two weeks post transfer and we did not see a large CAR T cell expansion in CAR+IL-15 group.

CAR T cells were detected in lymph nodes in the first therapeutic study, but they did not traffic there in the study with ART. The results indicate that CAR T cells require virus-producing cells to survive, but viral load suppression by cART may have limited the opportunity for CAR T cells to encounter target cells (i.e., latently infected cells in cART treated animals do not express sufficient viral Env on the cell surface for recognition). Co-transduction of CARs with a lymph node trafficking marker such as CXCR5 [31] might tissue trafficking of CAR T cells. Promising data were reported by Zhen et al. [32], who produced CAR cells using hematopoietic stem cells; these successfully differentiated into multilineage lymphocytes and CAR cells persisted more than two years. These data are in concert with our finding that using less differentiated cells would be an important to achieve viral control by CAR T cells.

A final hurdle to achieve viral control is low E:T ratio in vivo. Based on the in vitro data, the lowest E:T ratio to achieve viral suppression is 1:5. Virus infected cells are dispersed in lymph node when measured by in situ hybridization technologies [33]; achieving this level of effectors to targets seems difficult. Additionally, it should be noted that virus infected cells expressing Env protein for a relatively small fraction of the rapid viral lifecycle, and only towards the end when virus may be about to be released by cytolysis.

Overall, anti-SIV CAR T cell transfer in SIV-infected animals did not give either protection or viral control. Distinct from CAR T cell therapy in cancer, anti-HIV CAR therapy requires more improvement for viral control. However, our study informs CAR preparation in terms of target selection and in vitro expansion; these considerations will aid in any future application of this technology to HIV.

## Materials and methods

### Animal studies

This experiment utilized nonhuman primates (NHP) was performed in compliance with guidelines established by the Animal Welfare Act for laboratory animal housing and care and in accordance with a Vaccine Research Center (VRC) Institutional Animal Care and Use Committee approved animal study protocol. NHP studies were performed in ABSL-2 containment facilities at the National Institute of Health (NIH). The NIH is accredited by the Association for Assessment and Accreditation of Laboratory Animal Care (AAALAC) International.

All animals were Indian-origin rhesus macaques, male or female. Genotyping of MHC-I was performed and animals with MHC class I alleles Mamu-A01, -B08, or -B17 were excluded. For the SIV-CAR studies, animals were distributed into four groups and for the SHIV-CAR studies, animals were distributed into three groups of two for each of the non-ART and ART treatment arms. Groups were created based on the weight and the average weights were between 4.5-6kg.

For euthanasia: Animals were sedated with ketamine hydrochloride (10 mg/kg) IM and euthanized with Beuthanasia-D (390 mg pentobarbital sodium and 50 mg phenytoin/ml) at a dose of 2.2 ml/kg via IV route. Heartbeat and reparatory function were confirmed to be absent via aulsultation/palpation then a full necropsy was performed. For analgesia/anesthesia: for all procedures animals anesthetized with Ketamine (5–10 mg/kg) and Dexdomitor (0.01–0.03 mg/kg) IM. Animals were given Antisedan (0.5 mg/kg IM) post procedure as a reversal agent for the Dexdomitor. Analgesics were provided as outlined in the ASP and/or based on veterinary evaluation of the existance of pain. Analgesics approved for use on this study included Buprenorphine 0.03 mg/kg q12 hours) and Meloxicam (0.2 mg/kg SC/PO then 0.1 mg/kg SC/PO q24 hours).

All experimental procedures were overseen by veterinary staff, and were designed to minimize potential animal distress, pain and discomfort. Animals were pair-housed whenever possible to promote social species-specific behavior and were fed a standard species-appropriate diet adjusted for age and body weight. Food intake was monitored, and animals received food supplements and other enrichment devices daily. All animals were monitored by research and veterinary staff for criteria of humane endpoints, which included any condition resulting in untreatable pain or distress, or a poor prognosis for survival to the end of the study period. At the time points listed in the manuscript, animals were euthanized according to the recommendations of the American Veterinary Medical Association 2020 Panel on Euthanasia.

### SHIV and SIV-CAR constructs

SHIV-CAR constructs were similar to those as described previously [24]. The antigen binding moieties consisted of gp120-binding domains of CD4 alone or CD4 linked to a single chain variable region (scFv) of 17b with a 10 AA linker (CD4-10-17b) or 35 AA linker (CD4-35-17b). Intracellular domains of CD28 and CD3 zeta were then incorporated into the constructs. For SIV-CARs, scFv from SIV-specific neutralizing antibodies (SIVmAbs) were used for antigen binding moieties. We took scFv from CD4 binding site, V1/V2 and V3-specfic SIV mAbs. The transmembrane domain of the CD8α molecule was used in the SIV-CAR constructs. Intracellular domains of 4-1BB and CD3 zeta were incorporated into the constructs. Reporter genes, ZsGreen or NGFR, were designed to express from the internal ribosomal entry site following the CD3 zeta domain. All CARs were constructed on a gammaretrovirus backbone.

### Retrovirus vector production and transduction to T cells

Both SHIV and SIV-CARs were produced by transient transfection. For retrovirus production, backbone gammaretroviral DNA and RD114 env expressing DNA were co-transfected to GP2-293 cells using the Lipofectamine 2000 transfection reagent. Culture supernatant containing virions was collected at 48 and 72hrs after transfection and frozen down at -80°C. The transduction protocol was based on the published method [26]. Retroviral particles were attached to RetroNectin-coated plates. 0.5 x 106 rhesus PBMC pre-activated with IL-2 (200IU/ml) for 24hr were added to the coated plates and SHIV or SIV-CAR were transduced by centrifugation 2000 x g for 10 min at 32°C then incubated at 37°C overnight. On the next day, transduced cells were transferred to new virion-coated plates and the transduction process was repeated to increase transduction efficiency. In the dual SIV-CAR transduction, two distinct viral supernatants were mixed at 1:1 and used for plate coating. After transduction, cells were cultured in R-10 (IL-2: 200 IU/ml) at 37°C in 5% CO2 for more than 4 days and used for in vitro and in vivo experiments. For the SIV-CAR experiments, transduced CAR T cells were sorted on FACS Aria for in vitro experiments.

### SHIV-CAR T cell animal study

The design of the SHIV-CAR animal study is displayed in S2A Fig. The study included 12 animals total, 6 animals in the antiretroviral therapy arm and 6 in the non-antiretroviral therapy arm. In each of the arms, the 6 animals were split into groups of 2 which each group receiving either CD4, CD4-10-17b or CD4-35-17b CAR transduced cells. Every animal was infected with intravenous SHIV-162P3 virus 8wks prior to planned CAR T-cell transfusion. 4 wks prior to infusion, maximal blood was drawn from SHIV-infected animals for CAR T cell preparation. The 4wk ART regimen was started 2wk prior to CAR T cell transfer and was the same that was used in the SIV animal study described below. After initial infection, blood was sampled weekly to quantify viral load. After CAR T cell transfer, blood samples were collected daily for 1 wk and then weekly thereafter for 9 wks. BAL and inguinal LN biopsies were collected 1 wk post CAR T cell transfer. PBMCs were isolated from blood, LN biopsies and BAL and subsequently quantified and phenotyped as described below. One of the animals in the ART arm assigned to receive CD4-10-17b CAR T cells did not develop infection after challenge with IV virus and was excluded from further analysis.

### Preparation of SHIV-CAR T cells for adoptive transfer

Maximal blood (based on animal weight) was drawn from animals 4 wks prior to planned CAR T cell transfusion and PBMCs were isolated. SHIV-CAR T cells for autologous transfer were then prepared by two different methods–expanded or unexpanded. A portion of the isolated PBMCs were immediately transduced, sorted and expanded for 3 wks and thus termed “expanded.” The remainder of the cells were frozen and stored until 1 wk prior to transfusion when they were thawed and transfused without undergoing expansion and are thus termed “unexpanded.”

For the expanded cells, 1 wk after successful transduction with the SHIV-CAR constructs, transduced cells (which co-expressed the fluorescent marker, eGFP) were sorted on FACS Aria. Sorted cells were subsequently expanded using methods described previously [11] for 3 wks. Briefly cells were stimulated with 50ng/mL anti-CD3 (BD-Pharmingen Catalog # 551916, clone SP34-2), 50IU/mL IL-2 and irradiated human PBMC (irradiated with 6000 RAD) and human EBV-transformed B cell lines (irradiated with 15000 RAD). Every 2–3 days, 25–75% of the culture media was replaced with fresh media and 50IU/mL IL-2. Irradiated feeder cells were supplied again 2 wks later. For unexpanded cells, cells were thawed and transduced 1 wk prior to planned CAR T cell transfer without any further stimulation or sorting. Both expanded and unexpanded cells were phenotyped prior to transfer. Expanded and unexpanded cells were mixed at a 2:1 ratio and ~2e8 total cells were adoptively transferred to SHIV-infected animals.

### Anti-SHIV CAR T cell phenotyping

Surface markers and intracellular Ki67were stained for phenotyping and measuring in vivo adoptively transferred and proliferating anti-SHIV T Cells. After PBMCs were isolated from the blood and tissue samples, cells were washed twice with PBS and stained with LIVE/DEAD® Fixable Aqua Dead Cell Stain Kit (Thermo Fisher SCIENTIFIC) for 20 min at room temperature followed by staining with the following antibodies: CD3-Cy7-APC (Clone: SP34.2, BD Biosciences), CD4-BV605 (Clone: OKT4, BioLegend), CD8-BV711 (Clone: RPA-T8, BioLegend), CD28-Cy5PE (Clone: 28.2, BD Biosciences), CD45RA-Cy7-PE (Clone: L48, BD Pharmingen), CD69-ECD (Clone: TP1.55.3, Beckman Coulter), CCR7-Alexa Flour 680 (Clone: 150503, BD Pharmingen) and HLA-DR-BV421 (Clone: LN3, Biolegend). After surface staining, cells were permeabilized with Foxp3 / Transcription Factor Staining Buffer Set (eBiosciences) for 20 min at room temperature, then intracellular stained with anti-Ki67-PE (Clone: B56, BD Pharmingen).

### CAR T cell preparation to compare CAR T cell persistence under two different culture condition

In the comparison of CAR T cell *in vitro* expansion methods, maximum blood was drawn (based on animal weight) from two chronically SIVsmE660-FL14 infected rhesus macaques one month and five days before infusion. ITS01-CAR expressing ZsGreen was transduced into the isolated PBMC collected one month before infusion and ITS01-CAR expressing rhesus delta neural growth factor receptor (NGFR) was transduced into the PBMC isolated five days before *in vivo* transfer. Five days after ITS01-NGFR CAR transduction, ITS01-ZsGreen CAR T cells and ITS01-NGFR CAR T cells were mixed together and infused into the original monkeys. Anti-SIV CAR T cells were tracked by flow cytometry based on reporter gene expression.

### Prevention and therapeutic study by CAR T cells

In the therapeutic study, maximum blood was drawn from two chronically SIVsmE660-FL14-AK infected rhesus macaques five days before infusion and ITS01-ZsGreen CAR and ITS06-NGFR CAR were transduced into the isolated PBMC. Anti-SIV CAR T cells were maintained for 4 days then infused into the original animals.

In the prevention study, maximum blood was drawn from six SIV-negative rhesus macaques and ITS01-ZsGreen CAR T cells and ITS06-NGFR CAR T cells were transduced into isolated PBMC. Anti-SIV CAR T cells were maintained for 4 days *in vitro* and infused into the original animals followed by a SIVsmE660 intrarectal challenge at 10 TCID_50_. the control group received a SIVsmE660 intrarectal challenge only. After anti-SIV-CAR T cell infusion, blood was collected 1, 2 and 4 days post-infusion and weekly after the first week post-infusion. One week after infusion, left and right inguinal lymph node biopsies were performed and bronchoalveolar lavage was collected.

### Combination therapy with IL-15 and CAR T cells

24 animals were divided into 4 groups, which was sufficient to have an 80% probability of detecting at least a 1.5 Log_d_ difference in outcome viral load. ART was composed of subcutaneous 20 mg/Kg/day tenofovir (Gilead Sciences, CA, USA), 30 mg/Kg/day emtricitabine (Gilead Sciences, CA, USA) and 100 mg of raltegravir mixed with food and given twice a day (Merck, NJ, USA). ART started at wk 4 post SIV challenge and was terminated at wk 8. Triple CAR T cells were transferred at wk 7. 24 animals were divided into 4 groups. ART was composed of subcutaneous 20 mg/Kg/day tenofovir (Gilead Sciences, CA, USA), 30 mg/Kg/day emtricitabine (Gilead Sciences, CA, USA) and 100 mg of raltegravir mixed with food and given twice a day (Merck, NJ, USA). ART started at wk 4 post SIV challenge and was terminated at wk 8. Triple CAR T cells were transferred at wk 7. We combined CAR T cells transfer with delivery of IL-15. We employ the use of rhesus macaque heterodimeric IL-15 (rm hetIL-15), consisting of an heterodimer of the single chain IL-15 with the soluble region of IL-15Ra. hetIL-15 represent the natural form of IL-15 produced in the body [34, 35]. hetIL-15 was produced from human HEK293 cells and purified as previously described [36]. hetIL-15 was delivered SC twice/week for a total of 6 injections, following a dose escalation schedule, as previously reported [15, 37]. The initial dose was 13.5ug and the last dose was 153ug/monkey. Measurement of plasma IL-18 (Human IL-18 ELISA kit, MBL International Corporation) was performed at day 4 and 21, during the hetIL-15 cycle administration, as a measure of hetIL-15 bioactivity [15].

### SIV-CAR constructs

Single chain variable region (FcSv) from SIV-specific neutralizing antibodies (SIVmAbs) were used for antigen binding moieties. We took FcSv from CD4 binding site, V1/V2 and V3-specfic SIV mAbs. The transmembrane domain of the CD8α molecule was used in the SIV-CAR constructs. Intracellular domains of 4-1BB and CD3 zeta were incorporated into the constructs. Reporter genes, ZsGreen or NGFR, were designed to express from the internal ribosomal entry site following the CD3 zeta domain. SIV-CARs were constructed on a gammaretrovirus backbone.

### Retrovirus vector production and transduction to T cells

SIV-CARs were produced by transient transfection. For retrovirus production, backbone gammaretroviral DNA and RD114 env expressing DNA were co-transfected to GP2-293 cells using the Lipofectamine 2000 transfection reagent. Culture supernatant containing virions was collected at 48 and 72 hrs after transfection and frozen down at -80°C. The transduction protocol was based on the published method [24]. Retroviral particles were attached to RetroNectin-coated plates. 5 x 10^6^ rhesus PBMC pre-activated with IL-2 (200IU/ml) for 24 hr were added to the coated plates and SIV-CAR was transduced at 2000 x g, for 10 min at 32°C. On the next day, SIV-CAR transduced cells were transferred to new virion-coated plates and transduced again to increase transduction efficiency. In the dual SIV-CAR transduction, two or three distinct viral supernatants were mixed at equal volumes and used for plate coating. After transduction, cells were cultured in R-10 (IL-2: 200 IU/ml) at 37°C in 5% CO_2_ for more than 4 days and used for *in vitro* and *in vivo* experiments. SIV-CAR T cells were sorted on FACS Aria for *in vitro* experiments.

### Preparation of anti-SIV CAR T cells for in vivo studies

For the long-term expansion, ITS01-ZsGreen-transduced cells were stimulated with human PBMC and human EBV-transformed B cell lines irradiated with 6000 RAD and 15000 RAD using MARK I [^137^Cs] γ-irradiator. Feeder cells were supplied every 2 weeks and anti-SIV CAR T cells were maintained for one month. For the short-term expansion, ITS01-NGFR transduced cells were maintained in R-10 (IL-2: 200 IU/ml) for 4 days.

### In vitro viral suppression assay by anti-SIV CAR T cells

Viral suppression activity of anti-SIV CAR T cells was evaluated by co-culture with SIV-infected cells. CD8 T cells were depleted from rhesus PBMC using a CD8 MicroBead Kit, non-human primate (Miltenyi Biotec) and activated with ConcanavalinA (SIGMA Aldrich) for SIV infection. CD8-depleted rhesus PBMC were infected with tier 1, 2 or 3 SIV four days after activation. One day after infection, SIV-infected cells were washed three times and co-cultured with sorted anti-SIV CAR T cells. Intracellular p27 was stained or p27 concentration in the culture supernatant was measured three days after co-culture. Percentages of viral suppression were calculated based on changes in percentages of p27 positive cells or p27 concentration compared with the CAR T cell negative samples.

### Test of non-specific viral suppression by CAR negative cells after transduction

ZsGreen positive and negative CD4 and CD8 T cells were sorted on a FACS Aria after CAR transduction to rhesus PBMC and used as effector cells. On the day of co-culture, ZsGreen negative effector cells were stained with CellTrace™ Violet Cell Proliferation Kit (Thermo Fisher SCIENTIFIC) to be distinguished from target cells on a flow cytometer. Target cells infected with SIVsmE660-FL14-AK were co-cultured with CD4 or CD8 T cells or a combination of CD4 and CD8 T cells at E:T ratios of 1:1 or 1:1:1. Intracellular p27 was measured 4 days post-infection.

### Anti-SIV CAR binding to SIV gp140 probe

ZsGreen expressing anti-SIV CAR transduced cells were stained with anti-CD3-Cy7-APC, CD4-BV421, CD8-QDot655 and SIVmac239 gp140 Env trimer conjugated to PE. Cells were acquired on BD LSR II and the data were analyzed in FlowJo (Ashland, OR).

### Anti-SIV CAR T cell phenotyping

Surface markers and intracellular Ki67 were stained for phenotyping and measuring *in vivo* proliferation of anti-SIV CAR T cells. Rhesus PBMC were isolated. Cells were washed twice with PBS and stained with LIVE/DEAD® Fixable Aqua Dead Cell Stain Kit (Thermo Fisher SCIENTIFIC) for 20 min at room temperature followed by staining with the following antibodies: CD3-Cy7-APC (Clone: SP34.2, BD Biosciences), CD4-BV605 (Clone: OKT4, BioLegend), CD8-BV711 (Clone: RPA-T8, BioLegend), CD28-Cy5PE (Clone: 28.2, BD Biosciences), CD45RA-Cy7-PE (Clone: L48, BD Pharmingen), CD69-ECD (Clone: TP1.55.3, Beckman Coulter), CD95-APC (Clone: DX2, BD Biosciences), CCR7-Alexa Flour 700 (Clone: 150503, BD Pharmingen), HLA-DR-BV785 (Clone: L243, BioLegend), CD271 (Clone: C40-1457, BD Horizon). After surface staining, cells were permeabilized with Foxp3 / Transcription Factor Staining Buffer Set (eBiosciences) for 20 min at room temperature, then intracellular stained with anti-Ki67-PE (Clone: B56, BD Pharmingen). Cells were acquired on BD LSR II and BD FACSymphony.

For CAR+IL-15 combination therapy, we developed another flow panel for phenotyping. Dead cells were stained with LIVE/DEAD™ Fixable Blue Dead Cell Stain Kit, for UV excitation (Thermo Fisher). For surface staining, we used CD297-PerCP-eFlour710 (Clone: J105, eBioscience), CD45RA-Cy7PE (Clone: L48, BD Biosciences), CD28-Cy5-PE (Clone: 28.2, BioLegend), CXCR5-PE-eFlour610 (Clone: MU5UBEE, eBioscience), CD3-Cy7APC (Clone: SP34.2, BD Biosciences), MBL2-Alexa flour680 (3E5, LSBio), anti-ITS01-APC, HLA-DR-BV785 (Clone: L243, BioLegend), CD8-BV711 (Clone: RPA-T8, BioLegend), CD95-BV650 (Clone: DX2, BioLegend), CCR7-BV605 (Clone: 150503, BioLegend), NGFR-VioBlue (Clone: ME20.4-1.H4, Miltenyi), CD4-BUV661 (Clone: SK3, BD Horizon), CD69-BUV737 (Clone: FN50, BD Horizon). Ki67-BV480 (Clone: B56, BD Biosciences) was used for intra cellular staining after fixation/permeabilization. Cells were acquired on BD FACSymphony. All flow data were analyzed on Flowjo (BD Biosciences).

## Supporting information

S1 Fig SHIV and SIV-CAR constructs. SIV and SIV-CAR constructs. SHIV-CAR and CD4-MBL CAR had CD28 and CD3 zeta for signal transduction. To prevent virus infection to CAR-T cells, antigen binding moieties consist of domain 1 and 2 from CD4 molecule. For comparison of length of linker for target recognition, we used SHIV-ACR with 35AA and 10 AA linkers for CD4 and 17b CAR. SIVmAb CAR is designed to use 4-1BB and CD3 zeta signaling domain expecting better in vivo persistence resulting from maintenance of T_SCM_ and T_CM_.
(PDF)

S2 Fig Design of in vivo CAR studies in NHP. Design of in vivo CAR studies in NHP. (A) SHIV-CAR study outline. Animals were treated with/without ART for 4 weeks and CAR T cells were transferred 2 weeks post ART initiation. (B) Comparison of long-term and short-term expanded CAR T cells. PBMC isolated from maximum blood draw were stimulated with anti-CD3 and anti-CD28 antibodies for 3 days and transduced with ITS01-CAR expressing ZsGreen. ITS01-CAR T cells were expanded with irradiated human PBMC and BLCL. Short-term expanded CAR T cells were prepared from PBMC stimulated with IL-2. IL-2 stimulated PBMC were transduced with ITS01-CAR expressing NGFR and cultured for 4 days. ITS01-ZsGreen and ITS01-NGFR CAR T cells were mixed and transferred to the same animals simultaneously and compared in vivo persistence and memory phenotypes. (C) CAR-T cell therapy using dual CAR T cells. PBMC isolated from SIVsmE660-FL14 infected animals were transduced with ITS01-ZsGreen and ITS06.02-NGFR in the presence of IL-2. Dual CAR transduced PBMC were transferred to the original animals. (D) SIV prevention study using Dual CAR T cells. ITS01 and ITS06.02 CAR transduced PBMC were transferred at 1 day before SIV challenge.
(PDF)

S3 Fig Phenotype of expanded and unexpanded CAR T cells. Transduced T cells were expanded for 3 weeks (A) or minimally cultured (B). Flow cytometric assessment of the cells is shown: Progressive gating on live, CD3+, transduced (GFP+), CD4 or CD8 (top rows). The bottom rows show the differentiation stages as defined by expression of CCR7 and CD45RA, and activation status as defined by expression of CD69 and HLA-DR.
(PDF)

S4 Fig In vitro viral suppression activity on SIVmac239 and SIVsmE660-FL-14AK. In vitro viral suppression activity on SIVmac239 (A) and SIVsmE660-FL14-AK (B) by 7 different CAR T cells are evaluated. 139 CAR is control. CD4-MBL-ZsGreen, ITS01 and ITS06 CARs are transduced to triple CAR T cells. CAR T cells and SIV-infected target cells were co-cultured at E:T ratios of 1:1, 1:5, 1:25 and 1:125. Culture supernatants are collected on day 3, 5, 7, 10 and 13 post co-culture. p27 concentrations in the culture supernatants were measured by ELISA. Error bars represent the average and SD of triplicates.
(PDF)

S5 Fig Distribution of anti-SIV CAR memory phenotype. Percent of each memory fraction in CAR T cells at the time of transfer is demonstrated. T_SCM_: Blue, T_CM_: Green, T_EM_: Orange, T_TEM_: Red. Memory phenotypes are based on CCR and CD45RA expression. Each bar represents data from an animal.
(PDF)

S6 Fig Transition of CAR T cell memory phenotype after in vivo transfer and serum IL-18 levels in IL-15 treated animals. (A) Percent of each memory phenotype is shown in the graphs. Red: T_SCM_, yellow: T_CM_, green: T_EM_, blue: T_TEM_. (B) Plasma IL-18 measurement during hetIL-15 administration. Plasma IL-18 levels were determined at the indicated time points. Individual animals are shown.
(PDF)
