# Supplementary material for: Evaluation of chimeric antigen receptor T cell therapy in non-human primates infected with SHIV or SIV
Source: PLoS One. 2021 Mar 22;16(3):e0248973. doi: 10.1371/journal.pone.0248973 (PMC7984852; doi:10.1371/journal.pone.0248973)
Supplement: S1 Fig — SIV and SIV-CAR constructs. SHIV-CAR and CD4-MBL CAR had CD28 and CD3 zeta for signal transduction. To prevent virus infection to CAR-T cells, antigen binding moieties consist of domain 1 and 2 from CD4 molecule. For comparison of length of linker for target recognition, we used SHIV-ACR with 35AA and 10 AA linkers for CD4 and 17b CAR. SIVmAb CAR is designed to use 4-1BB and CD3 zeta signaling domain expecting better in vivo persistence resulting from maintenance of TSCM and TCM. (PDF) [file pone.0248973.s001.pdf]

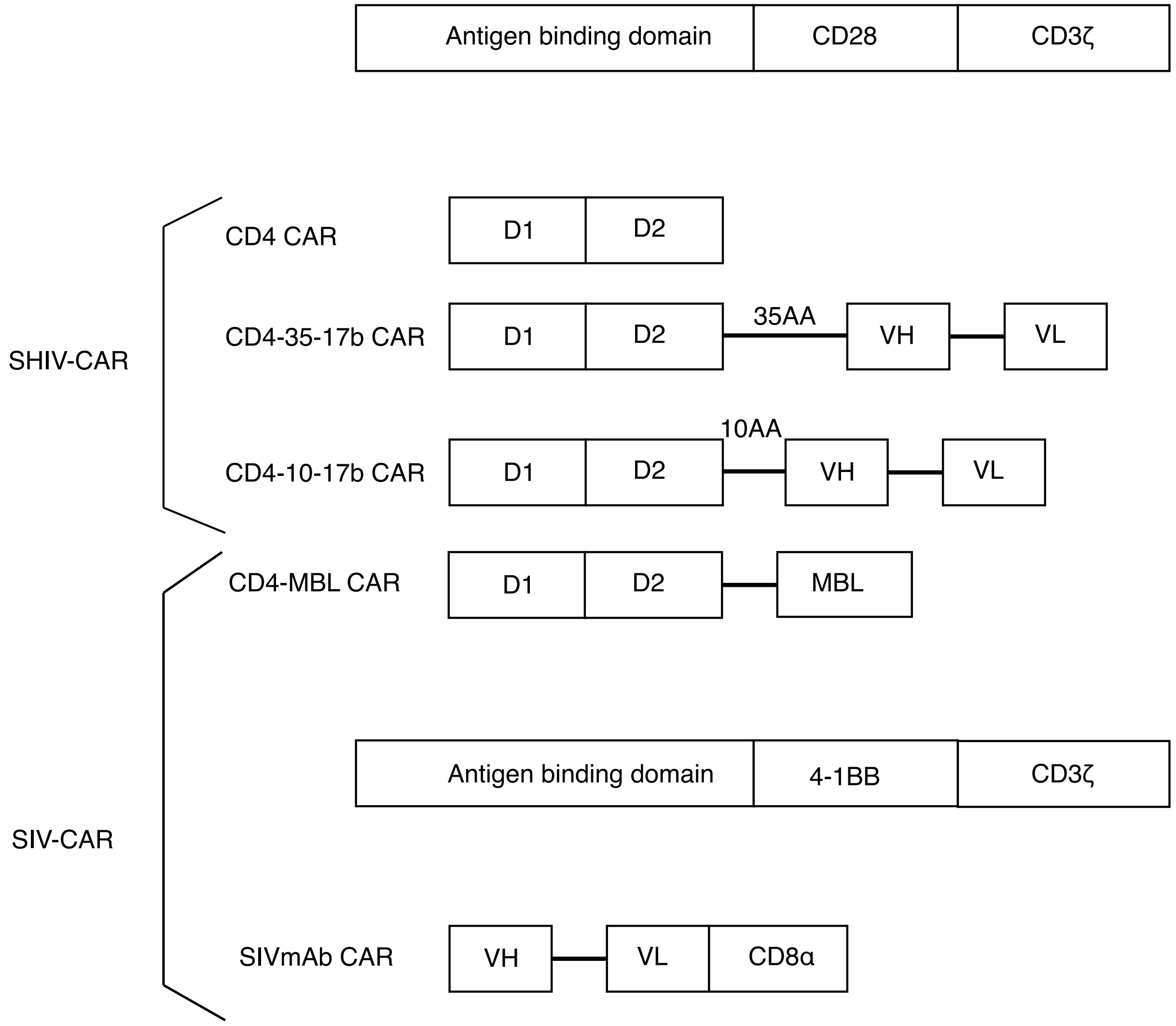

S1 Fig. SHIV and SIV-CAR constructs. SIV and SIV-CAR constructs. SHIV-CAR and CD4-MBL CAR had CD28 and CD3 zeta for signal transduction. To prevent virus infection to CAR-T cells, antigen binding moieties consist of domain 1 and 2 from CD4 molecule. For comparison of length of linker for target recognition, we used SHIV-ACR with 35AA and 10 AA linkers for CD4 and 17b CAR. SIVmAb CAR is designed to use 4-1BB and CD3 zeta signaling domain expecting better in vivo persistence resulting from maintenance of T_{SCM} and T_{CM}.
