# Supplementary material for: Evaluation of chimeric antigen receptor T cell therapy in non-human primates infected with SHIV or SIV
Source: PLoS One. 2021 Mar 22;16(3):e0248973. doi: 10.1371/journal.pone.0248973 (PMC7984852; doi:10.1371/journal.pone.0248973)
Supplement: S2 Fig — Design of in vivo CAR studies in NHP. (A) SHIV-CAR study outline. Animals were treated with/without ART for 4 weeks and CAR T cells were transferred 2 weeks post ART initiation. (B) Comparison of long-term and short-term expanded CAR T cells. PBMC isolated from maximum blood draw were stimulated with anti-CD3 and anti-CD28 antibodies for 3 days and transduced with ITS01-CAR expressing ZsGreen. ITS01-CAR T cells were expanded with irradiated human PBMC and BLCL. Short-term expanded CAR T cells were prepared from PBMC stimulated with IL-2. IL-2 stimulated PBMC were transduced with ITS01-CAR expressing NGFR and cultured for 4 days. ITS01-ZsGreen and ITS01-NGFR CAR T cells were mixed and transferred to the same animals simultaneously and compared in vivo persistence and memory phenotypes. (C) CAR-T cell therapy using dual CAR T cells. PBMC isolated from SIVsmE660-FL14 infected animals were transduced with ITS01-ZsGreen and ITS06.02-NGFR in the presence of IL-2. Dual CAR transduced PBMC were transferred to the original animals. (D) SIV prevention study using Dual CAR T cells. ITS01 and ITS06.02 CAR transduced PBMC were transferred at 1 day before SIV challenge. (PDF) [file pone.0248973.s002.pdf]

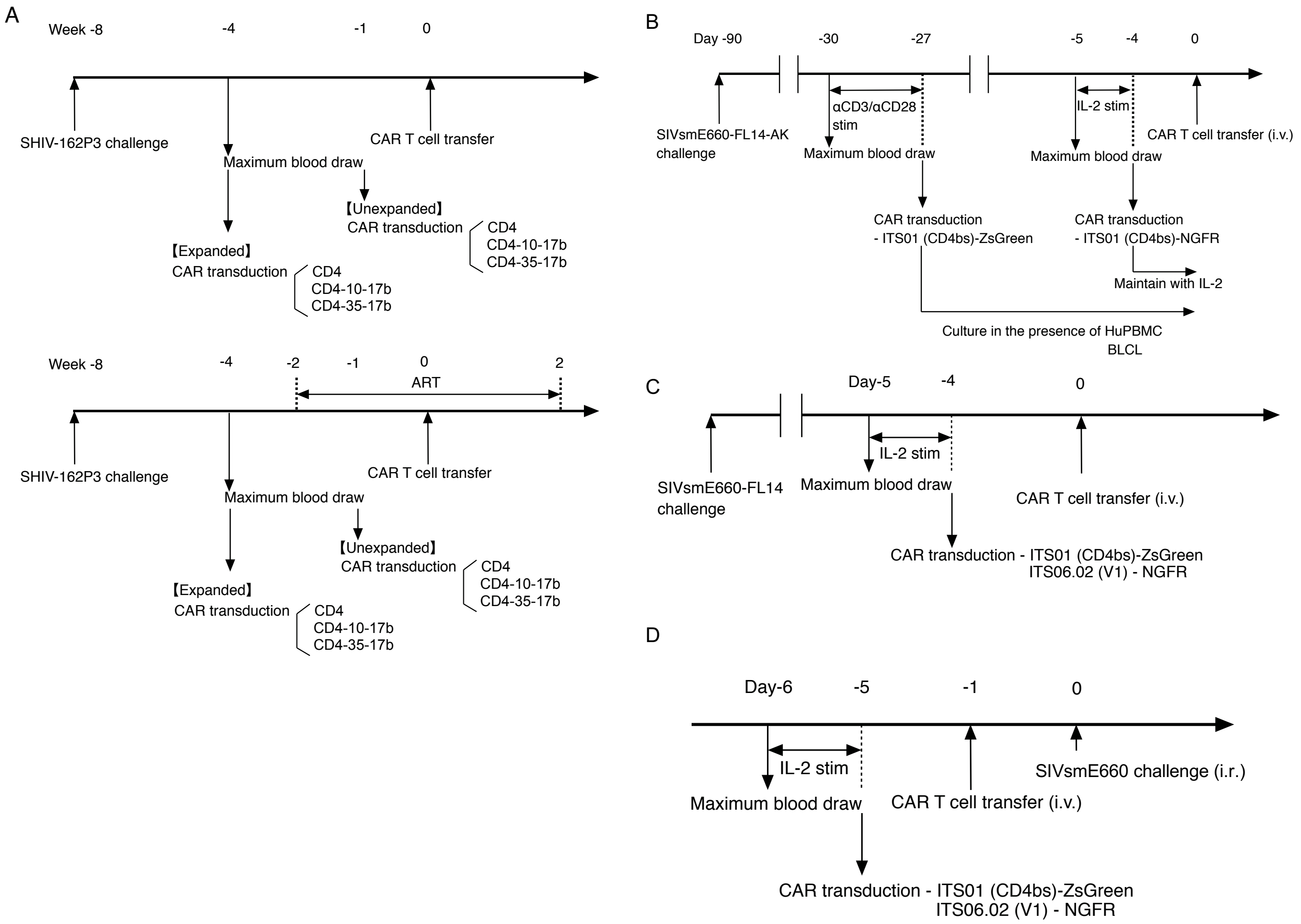

S2 Fig. Design of in vivo CAR studies in NHP. Design of in vivo CAR studies in NHP. (A) SHIV-CAR study outline. Animals were treated with/without ART for 4 weeks and CAR T cells were transferred 2 weeks post ART initiation. (B) Comparison of long-term and short-term expanded CAR T cells. PBMC isolated from maximum blood draw were stimulated with anti-CD3 and anti-CD28 antibodies for 3 days and transduced with ITS01-CAR expressing ZsGreen. ITS01-CAR T cells were expanded with irradiated human PBMC and BLCL. Short-term expanded CAR T cells were prepared from PBMC stimulated with IL-2. IL-2 stimulated PBMC were transduced with ITS01-CAR expressing NGFR and cultured for 4 days. ITS01-ZsGreen and ITS01-NGFR CAR T cells were mixed and transferred to the same animals simultaneously and compared in vivo persistence and memory phenotypes. (C) CAR-T cell therapy using dual CAR T cells. PBMC isolated from SIVsmE660-FL14 infected animals were transduced with ITS01-ZsGreen and ITS06.02-NGFR in the presence of IL-2. Dual CAR transduced PBMC were transferred to the original animals. (D) SIV prevention study using Dual CAR T cells. ITS01 and ITS06.02 CAR transduced PBMC were transferred at 1 day before SIV challenge.
