# Supplementary material for: Evaluation of chimeric antigen receptor T cell therapy in non-human primates infected with SHIV or SIV
Source: PLoS One. 2021 Mar 22;16(3):e0248973. doi: 10.1371/journal.pone.0248973 (PMC7984852; doi:10.1371/journal.pone.0248973)
Supplement: S3 Fig — Transduced T cells were expanded for 3 weeks (A) or minimally cultured (B). Flow cytometric assessment of the cells is shown: Progressive gating on live, CD3+, transduced (GFP+), CD4 or CD8 (top rows). The bottom rows show the differentiation stages as defined by expression of CCR7 and CD45RA, and activation status as defined by expression of CD69 and HLA-DR. (PDF) [file pone.0248973.s003.pdf]

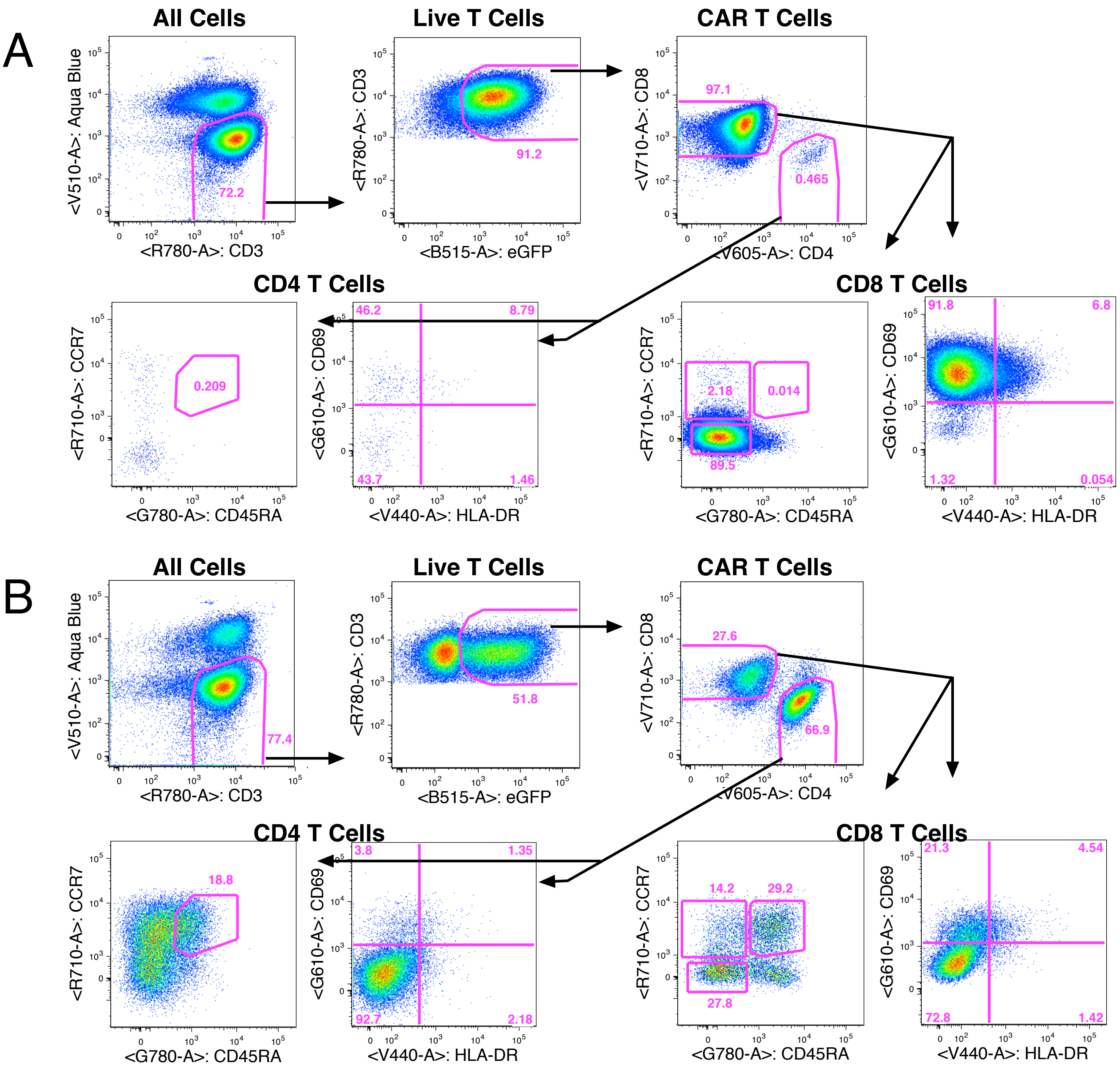

S3 Fig. Phenotype of expanded and unexpanded CAR T cells. Transduced T cells were expanded for 3 weeks (A) or minimally cultured (B). Flow cytometric assessment of the cells is shown: Progressive gating on live, CD3+, transduced (GFP+), CD4 or CD8 (top rows). The bottom rows show the differentiation stages as defined by expression of CCR7 and CD45RA, and activation status as defined by expression of CD69 and HLA-DR.
