# Supplementary material for: Evaluation of chimeric antigen receptor T cell therapy in non-human primates infected with SHIV or SIV
Source: PLoS One. 2021 Mar 22;16(3):e0248973. doi: 10.1371/journal.pone.0248973 (PMC7984852; doi:10.1371/journal.pone.0248973)
Supplement: S4 Fig — In vitro viral suppression activity on SIVmac239 (A) and SIVsmE660-FL14-AK (B) by 7 different CAR T cells are evaluated. 139 CAR is control. CD4-MBL-ZsGreen, ITS01 and ITS06 CARs are transduced to triple CAR T cells. CAR T cells and SIV-infected target cells were co-cultured at E:T ratios of 1:1, 1:5, 1:25 and 1:125. Culture supernatants are collected on day 3, 5, 7, 10 and 13 post co-culture. p27 concentrations in the culture supernatants were measured by ELISA. Error bars represent the average and SD of triplicates. (PDF) [file pone.0248973.s004.pdf]

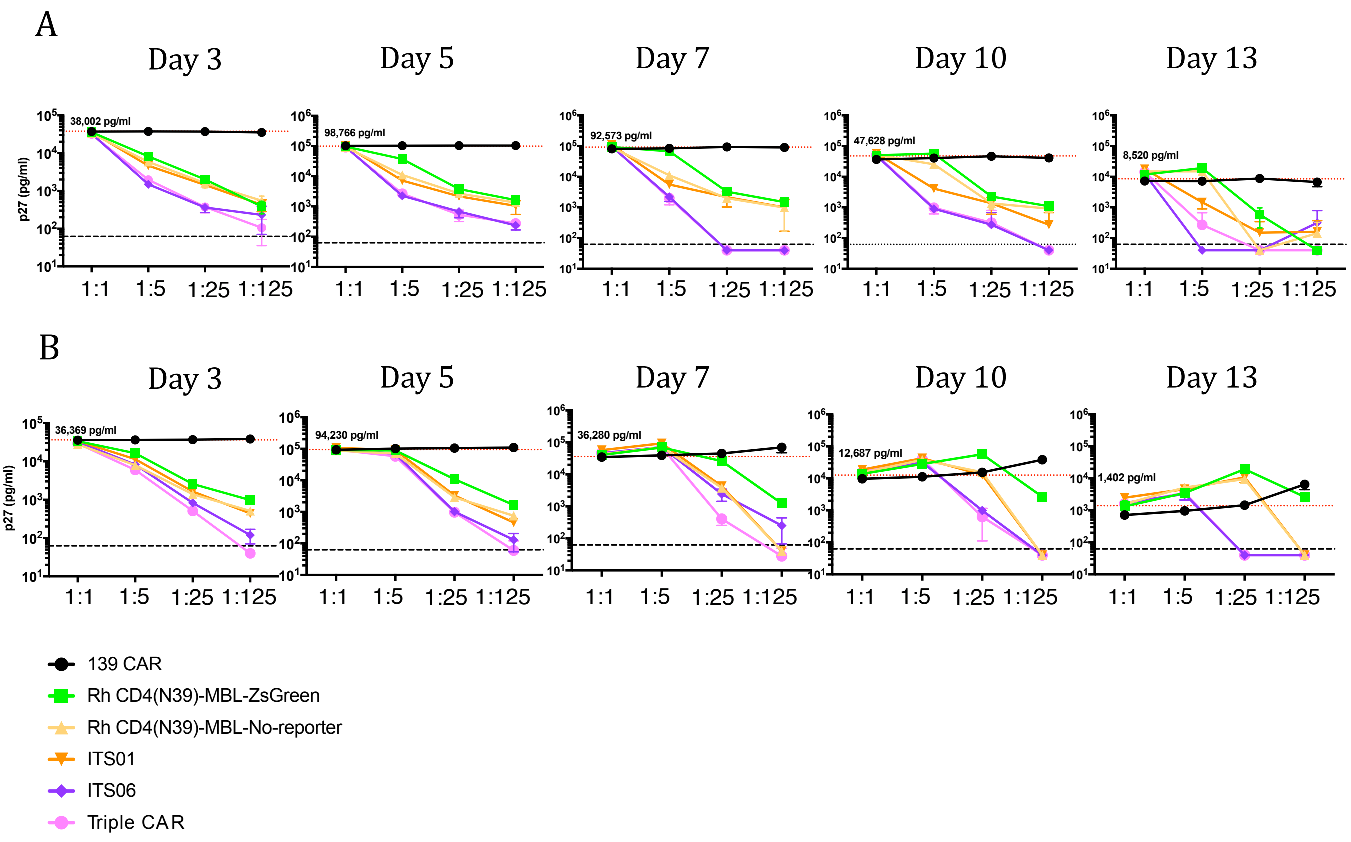

S4 Fig. In vitro viral suppression activity on SIVmac239 and SIVsmE660-FL-14AK. In vitro viral suppression activity on SIVmac239 (A) and SIVsmE660-FL14-AK (B) by 7 different CAR T cells are evaluated. 139 CAR is control. CD4-MBL-ZsGreen, ITS01 and ITS06 CARs are transduced to triple CAR T cells. CAR T cells and SIV-infected target cells were co-cultured at E:T ratios of 1:1, 1:5, 1:25 and 1:125. Culture supernatants are collected on day 3, 5, 7, 10 and 13 post co-culture. p27 concentrations in the culture supernatants were measured by ELISA. Error bars represent the average and SD of triplicates.
