# Supplementary material for: Evaluation of chimeric antigen receptor T cell therapy in non-human primates infected with SHIV or SIV
Source: PLoS One. 2021 Mar 22;16(3):e0248973. doi: 10.1371/journal.pone.0248973 (PMC7984852; doi:10.1371/journal.pone.0248973)
Supplement: S6 Fig — (A) Percent of each memory phenotype is shown in the graphs. Red: TSCM, yellow: TCM, green: TEM, blue: TTEM. (B) Plasma IL-18 measurement during hetIL-15 administration. Plasma IL-18 levels were determined at the indicated time points. Individual animals are shown. (PDF) [file pone.0248973.s006.pdf]

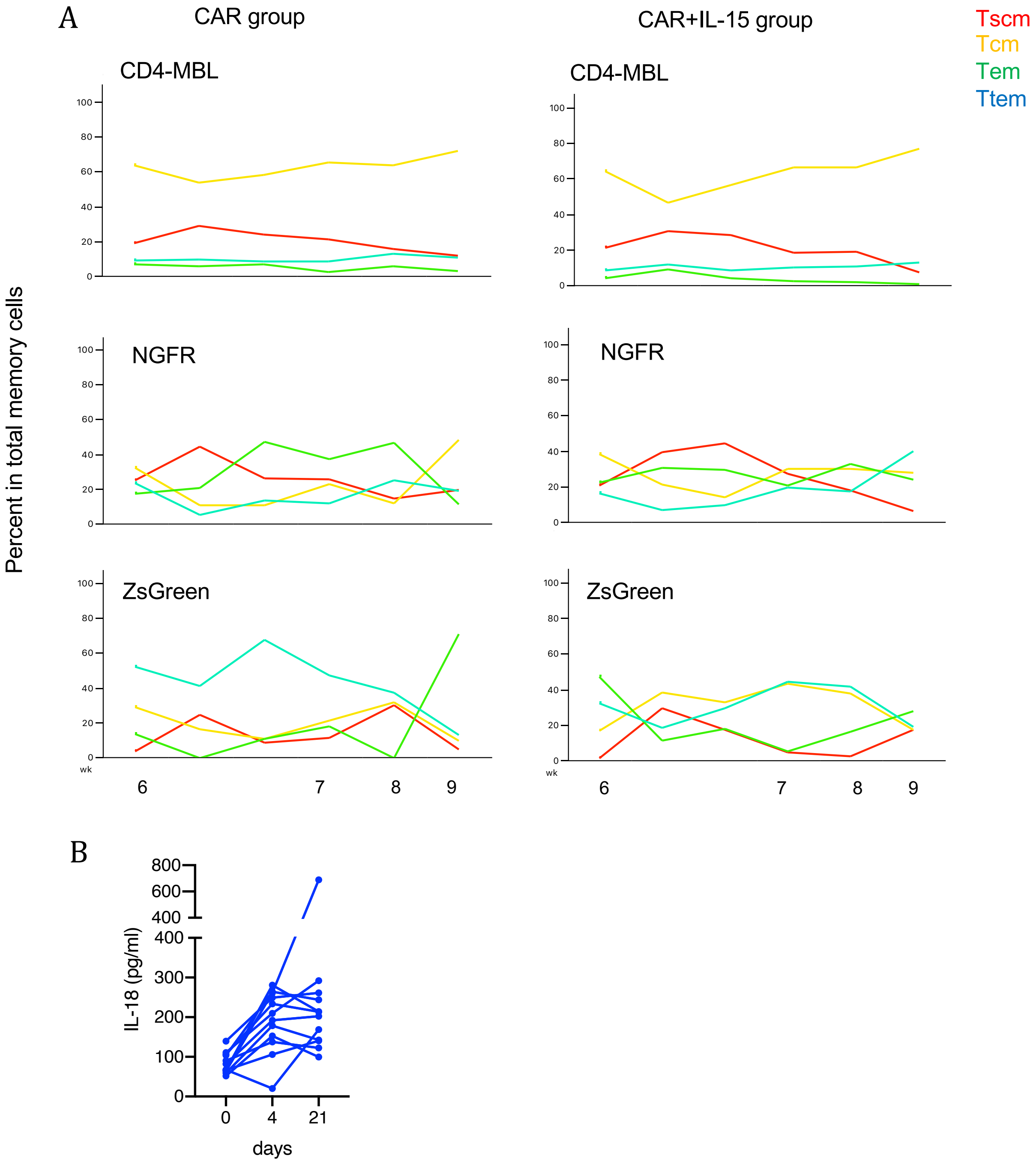

S6 Fig. Transition of CAR T cell memory phenotype after in vivo transfer and serum IL-18 levels in IL-15 treated animals. (A) Percent of each memory phenotype is shown in the graphs. Red: T_{SCM}, yellow: T_{CM}, green: T_{EM}, blue: T_{TEM}. (B) Plasma IL-18 measurement during hetIL-15 administration. Plasma IL-18 levels were determined at the indicated time points. Individual animals are shown.
