# Supplementary material for: Evaluation of chimeric antigen receptor T cell therapy in non-human primates infected with SHIV or SIV
Source: PLoS One. 2021 Mar 22;16(3):e0248973. doi: 10.1371/journal.pone.0248973 (PMC7984852; doi:10.1371/journal.pone.0248973)
Supplement: S5 Fig — Percent of each memory fraction in CAR T cells at the time of transfer is demonstrated. TSCM: Blue, TCM: Green, TEM: Orange, TTEM: Red. Memory phenotypes are based on CCR and CD45RA expression. Each bar represents data from an animal. (PDF) [file pone.0248973.s005.pdf]

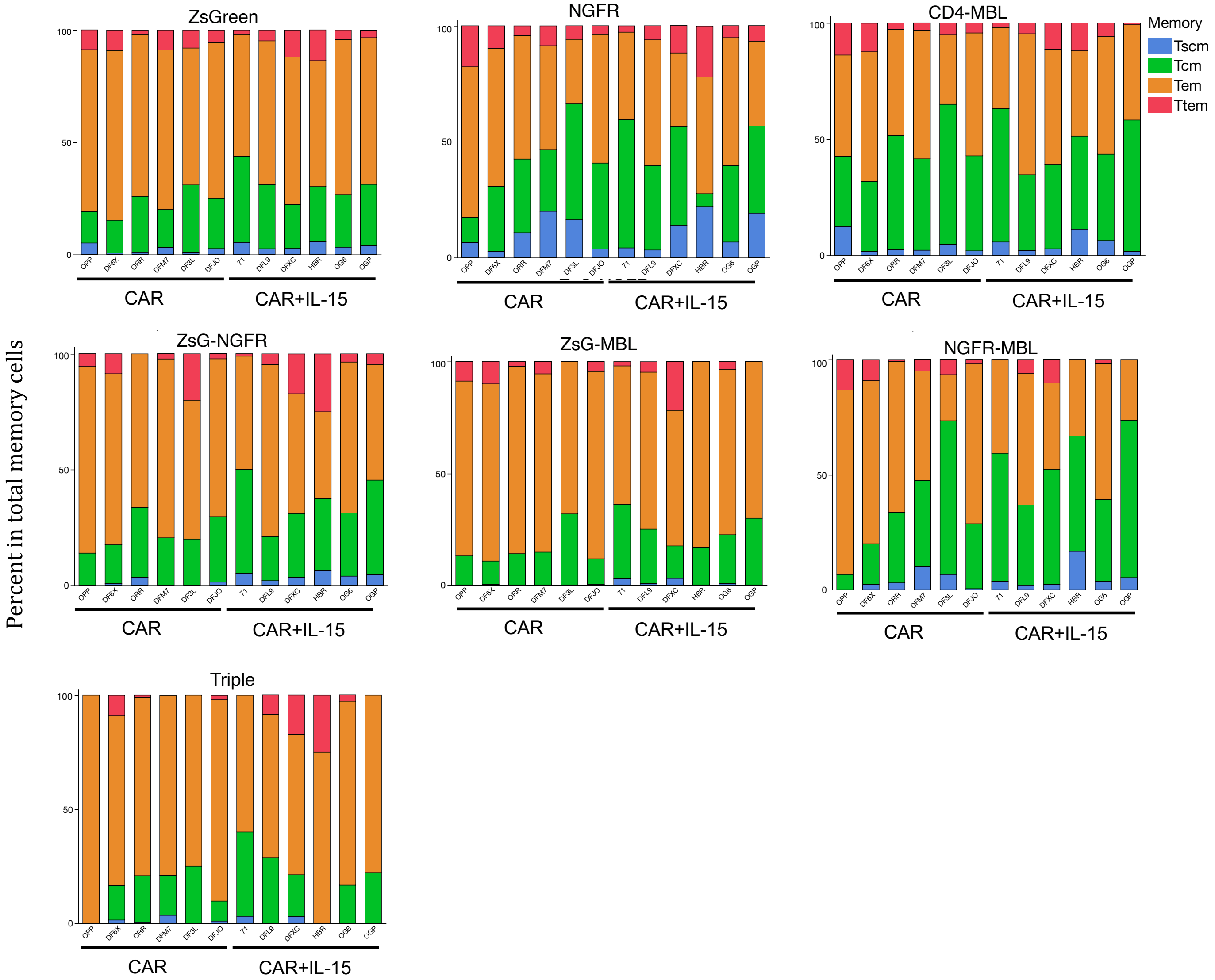

S5 Fig. Distribution of anti-SIV CAR memory phenotype. Percent of each memory fraction in CAR T cells at the time of transfer is demonstrated. T_{SCM}: Blue, T_{CM}: Green, T_{EM}: Orange, T_{TEM}: Red. Memory phenotypes are based on CCR and CD45RA expression. Each bar represents data from an animal.
